# Innovations in WO_3_ gas sensors: Nanostructure engineering, functionalization, and future perspectives

**DOI:** 10.1016/j.heliyon.2024.e27740

**Published:** 2024-03-13

**Authors:** Xingxing Li, Li Fu, Hassan Karimi-Maleh, Fei Chen, Shichao Zhao

**Affiliations:** a Key Laboratory of Novel Materials for Sensor of Zhejiang Province, College of Materials and Environmental Engineering, Hangzhou Dianzi University, Hangzhou, 310018, PR China; b School of Resources and Environment, University of Electronic Science and Technology of China, 611731, Chengdu, PR China; c School of Engineering, Lebanese American University, Byblos, Lebanon

**Keywords:** Tungsten oxide, Gas sensors, Nanostructuring, p-n heterojunctions, UV activation, Sensitivity and selectivity enhancement

## Abstract

This review critically examines the progress and challenges in the field of nanostructured tungsten oxide (WO_3_) gas sensors. It delves into the significant advancements achieved through nanostructuring and composite formation of WO_3_, which have markedly improved sensor sensitivity for gases like NO_2_, NH_3_, and VOCs, achieving detection limits in the ppb range. The review systematically explores various innovative approaches, such as doping WO_3_ with transition metals, creating heterojunctions with materials like CuO and graphene, and employing machine learning models to optimize sensor configurations. The challenges facing WO_3_ sensors are also thoroughly examined. Key issues include cross-sensitivity to different gases, particularly at higher temperatures, and long-term stability affected by factors like grain growth and volatility of dopants. The review assesses potential solutions to these challenges, including statistical analysis of sensor arrays, surface functionalization, and the use of novel nanostructures for enhanced performance and selectivity. In addition, the review discusses the impact of ambient humidity on sensor performance and the current strategies to mitigate it, such as composite materials with humidity shielding effects and surface functionalization with hydrophobic groups. The need for high operating temperatures, leading to higher power consumption, is also addressed, along with possible solutions like the use of advanced materials and new transduction principles to lower temperature requirements. The review concludes by highlighting the necessity for a multidisciplinary approach in future research. This approach should combine materials synthesis, device engineering, and data science to develop the next generation of WO_3_ sensors with enhanced sensitivity, ultrafast response rates, and improved portability. The integration of machine learning and IoT connectivity is posited as a key driver for new applications in areas like personal exposure monitoring, wearable diagnostics, and smart city networks, underlining WO_3_'s potential as a robust gas sensing material in future technological advancements.

## 1 Introduction

Gas sensors play an indispensable role in monitoring and assessing air quality in various indoor and outdoor environments. The ability to reliably detect and quantify concentrations of toxic, hazardous, and combustible gases is critical for applications ranging from air pollution monitoring to industrial safety and medical diagnostics. Metal oxide semiconductors have emerged as one of the most promising sensing materials for gas sensor technologies due to their low cost, simple fabrication, high sensitivity and stability [1,2,2,3]. Within this class of metal oxide sensors, WO_3_ has attracted significant research interest owing to its excellent gas sensing capabilities [4,5].

WO_3_ is an n-type semiconductor metal oxide that has been widely explored for resistive-type gas sensors. p-type WO_3_ can be achieved through doping with elements like Na, K, Mg, Zn, Fe, Sb, and C [[6], [7], [8]]. The valence states of these dopants provide holes that act as majority charge carriers, transforming the conduction to p-type. Compared to n-type WO_3_, p-type WO_3_ sensors tend to operate at lower temperatures from room temperature to around 200 °C. WO_3_ has a band gap of 2.6–2.8 eV and its conduction is attributed to electrons [9]. Nanostructured WO_3_ often shows bandgap values on the lower end near 2.6 eV, while bulk and thin film WO_3_ is usually above 3.0 eV [10]. The bandgaphas implications for electrical conductivity, optical absorption, photocatalysis, and photoactivation effects [11]. For gas sensing, smaller bandgaps can enable room temperature activation but may impact selectivity [12]. Larger bandgaps improve stability but increase the operating temperature. So there is a tradeoff that depends on the specific application. WO_3_ demonstrates excellent sensitivity to both oxidizing gases such as nitrogen dioxide (NO_2_), sulfur dioxide (SO_2_), ozone (O_3_) as well as reducing gases like carbon monoxide (CO), ammonia (NH_3_), hydrogen sulfide (H_2_S) and volatile organic compounds (VOCs) [13]. The gas sensing mechanism relies on the adsorption and reaction of target gas molecules with negatively charged oxygen species on the WO_3_ surface [14]. This modulates the electron concentration in WO_3_, thereby changing its electrical conductivity. For oxidizing gases, electrons are extracted from the conduction band leading to increased resistance [[15], [16], [17], [18], [19]]. Reducing gases inject electrons into the conduction band and cause decreased resistance [13]. The sensitivity depends on factors like operating temperature, morphology, exposed facets and oxygen vacancies [14].

Various techniques have been employed to synthesize WO_3_ nanostructures for gas sensing, including wet chemical approaches like hydrothermal, sol-gel, precipitation and combustion methods as well as dry techniques such as sputtering, thermal evaporation and pulsed laser deposition [20]. The morphology can be controlled at the nanoscale to obtain different dimensionalities like 0D nanoparticles, 1D nanowires/nanorods, 2D nanosheets and 3D hierarchical structures [21]. Lower dimensional nanostructures help enhance the surface area and gas accessibility. Modified WO_3_ with exposed high energy crystal facets also demonstrate improved gas interaction. In addition, elemental doping, noble metal functionalization, composite formation and heterostructuring with other nanomaterials have been shown to significantly boost sensitivity and response kinetics [22].

Owing to its good stability, reproducibility and high sensitivity at low temperatures, WO_3_ has emerged as a promising sensing material for portable and wearable gas sensor devices [23]. It has been applied for environmental monitoring of pollutants such as NO_2_, SO_2_, VOC's and for detection of toxic industrial gases. Inside vehicles, WO_3_ sensors can detect CO and alcohol vapors for air quality control [24]. They also have uses in medical diagnostics for breath analysis by sensing biomarkers like acetone, ammonia and hydrogen [23,25,26]. Within petrochemical industry, WO_3_ sensors enable leakage monitoring of combustion gases [27]. Thus WO_3_ gas sensors have diverse applications across automation, safety, security, biomedical, food and agriculture sectors. However, most WO_3_ gas sensors continue to suffer from issues like low selectivity, humidity interference and long-term drift which limit their reliability and commercial viability [14]. Typical approaches to enhance selectivity include use of filters, sensor arrays, and multivariate data analysis. But these increase system cost and complexity [13]. Stability and lifetime is affected by factors like grain growth, sintering and component volatility at operating temperatures [13]. So new nanostructures and composites need to be explored to minimize these degradation mechanisms. There is also a need to develop low power WO_3_ sensors that can operate at room temperature or with minimal heating. This can be enabled by investigating new physical or chemical activation techniques.

WO_3_ gas sensors have drawn significant interest for their potential to enable impactful real-world applications in areas such as air quality monitoring, wearable medical diagnostics, industrial safety, and food/agricultural technology. However, translating WO_3_ capabilities into field deployments requires tackling key challenges around sensitivity, selectivity, stability, and system integration. For instance, there is a growing need for WO_3_ sensors with part-per-trillion sensitivities to facilitate early disease detection through breath analysis, requiring innovation in materials and surface engineering. Enhancing selectivity in complex gas mixtures is also critical for reliable environmental sensing and household safety applications. Moreover, stability improvements would expand biomonitoring and infrastructure maintenance uses. While there have been a number of recent reviews on nanostructured WO_3_ gas sensors [[28], [29], [30], [31]], our work stands out in its comprehensive yet incisive analysis from materials design to device engineering to reliability assessments. Specifically, we provide updated perspectives on.

(i) Broad evaluation of synthetic techniques spanning wet chemical and vapor deposition methods to achieve fine morphology control not covered to such extent before,
(ii) Systematic analysis of doping, heterojunctions and facet engineering for performance enhancement across sensitivity, selectivity and kinetics through tabulated examples
(iii) Target gas analyte breadth encompassing environmental pollutants, industrial gases, combustibles and medical biomarkers, towards highlighting emerging applications
(iv) Critical assessment of intrinsic limitations around stability, humidity tolerance, baseline drift and power consumption, along with mitigation strategies and future research directions

## 2 WO_3_ gas sensing mechanism

The underlying gas sensing mechanism in WO_3_ relies on changes in its electrical conductivity when exposed to target gas molecules. WO_3_ is an n-type semiconductor with a bandgap of 2.6–2.8 eV. Its electrical conduction is attributed to electrons excited into the conduction band from the valence band [32]. The gas sensing properties arise from modulation of electron concentration upon interaction with analyte gas molecules [33].

In ambient air, oxygen is adsorbed on the surface of WO_3_ and captures electrons from the conduction band:

O_2_(gas) + e^−^ → O_2_^−^ (adsorbed)

This leaves behind positively charged donors and creates an electron-depleted region on the WO_3_ surface. The thickness of this charged layer depends on the temperature and concentration of ambient oxygen. Higher temperatures provide the kinetic energy needed to accelerate the adsorption and ionization of oxygen molecules. This reaction with surface oxygen species causes electron depletion in the WO_3_ surface and upward band bending [34].

The negatively charged oxygen ions can further capture electrons from the conduction band to form superoxide (O_2_^−^) and peroxide (O_2_^2−^) ions:

O_2_^−^ + e^−^ → 2O^−^

O^−^ + e^−^ → O_2_^−^

The type of oxygen species present on the WO_3_ surface depends on the operating temperature. Below 100 °C, the O_2_^−^ ion is predominant. In the range of 100–300 °C, O^−^ ions dominate, while above 300 °C, O_2_^−^ becomes the major species. The electronegativity of these oxygen ions makes them reactive to both oxidizing and reducing gases [35].

When WO_3_ is exposed to an oxidizing gas like NO_2_, the gas molecules capture electrons from the conduction band:

NO_2_ + e^−^ → NO_2_^−^ (adsorbed)

This additional electron depletion by the adsorbed NO_2_^−^ causes increased band bending and resistance of the WO_3_ sensor. For reducing gases like CO, NH_3_, H_2_S or VOCs, the gas molecules react with the negatively charged oxygen species:

CO + O^−^ → CO_2_ + e^-^

This releases the trapped electrons back to the WO_3_ conduction band, thereby decreasing the resistance. The sensitivity is defined as R_a_/R_g_, where R_a_ and R_g_ are the resistances in air and target gas respectively. For n-type oxides like WO_3_, oxidizing gases increase R_a_ and cause a positive sensitivity, while reducing gases reduce R_g_ and give a negative sensitivity [36].

The gas response is also affected by the operating temperature. For WO_3_, the optimal temperature is typically 100–400 °C [37]. At 100–300 °C, the oxygen adsorption rate increases by up to 2× compared to room temperature, leading to a corresponding increase in gas response. As the temperature rises from 300 to 400 °C, the gas response continues improving but at a slower rate due to the counteracting impact of initial sintering. Above 400 °C, the gas response declines dramatically (by as much as 10× compared to peak value) because of accelerated sintering and grain growth that reduce the active surface area and gas diffusion pathways (Table 1.).

**Table 1:** Relative change in key parameters affecting gas response at different temperatures.

Temperature (°C)	Oxygen Adsorption Rate	Gas Response	Reason
100–300	Increases up to 2×	Increases up to 2×	Enhanced O_2_ adsorption [38]
**300–400**	Slower increase	Continued increase but slower rate	Initial sintering counteracts O_2_ adsorption
**>400**	–	Declines by up to 10×	Accelerated sintering reduces surface area [29]

**Fig. 1:**
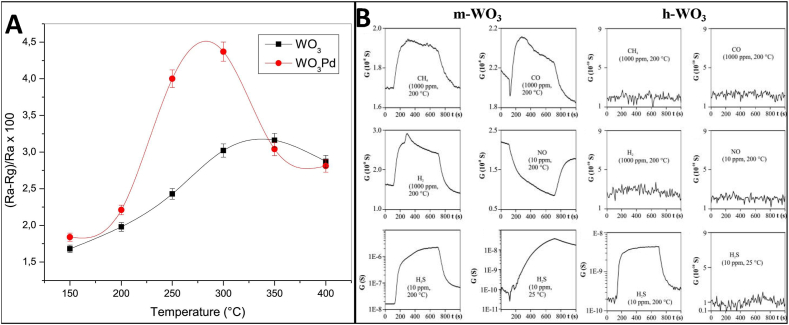
(A) Sensor response of annealed unloaded and 10% mol Pd loaded WO_3_ films to 50 ppm NH_3_ vs. operating temperature [40]. (B) Gas sensitivity of m-WO_3_ and h-WO_3_ to CH_4_ (1000 ppm, 200 °C); CO (1000 ppm, 200 °C); H_2_ (1000 ppm, 200 °C); NO (10 ppm, 200 °C); H_2_S (10 ppm, 200 °C) and H_2_S (10 ppm, 25 °C) [41]. Permission obtained from ELSEVIER.

In addition to temperature, the gas response depends on microstructural factors like grain size, porosity, crystal structure, exposed facets, oxygen vacancies and doping [29]. Elements like Pt, Pd, Au dopants further catalyze the dissociation of oxygen, thereby enhancing sensitivity [39]. For example, the ammonia sensing properties of the Pd-loaded WO_3_ films were evaluated at different operating temperatures [40]. It was found that the 10% Pd– WO_3_ films showed an excellent response down to 50 ppm of NH_3_ at an optimal operating temperature of 300 °C. Specifically, these films exhibited a sensitivity of around 60% towards 50 ppm NH_3_. The sensitivity is defined as the relative change in electrical resistance upon exposure to the target gas. In comparison, pure WO_3_ films without Pd loading showed negligible response at this low NH_3_ concentration. The Pd functionalization significantly enhanced the response and recovery kinetics of the WO_3_ sensor. The 10% Pd-WO_3_ films demonstrated response and recovery times of around 100 s, which were nearly 2–3 times faster than pure WO_3_ films (Fig. 1A).

Monoclinic and hexagonal phases of WO_3_ demonstrated different sensing performance [42,41]. As shown in Fig. 1B, the gas sensing tests showed monoclinic WO_3_ (m-WO_3_) could detect CH_4_, CO, H_2_, NO and H_2_S at 200 °C, with the highest sensitivity to 10 ppm H_2_S resulting in a conductivity increase over 100 times greater than for the other gases. m-WO_3_ could even detect H_2_S at room temperature. In contrast, hexagonal WO_3_ (h-WO_3_) only responded to 10 ppm H_2_S at 200 °C, with a smaller conductivity increase compared to m-WO_3_. However, the response time of h-WO_3_ to H_2_S was faster. While m-WO_3_ exhibited relative selectivity for H_2_S over the other gases, h-WO_3_ displayed absolute selectivity only responding to H_2_S. The crystal structure of WO_3_ clearly impacts gas selectivity, with the hexagonal polymorph providing superior selectivity for H_2_S detection. This demonstrates tuning the crystal structure of metal oxides like WO_3_ can improve selectivity, which is important for practical gas sensor applications.

High energy crystal facets like (002) improve gas adsorption compared to (100) facets [43]. Liang et al. [44] synthesized an ultrathin WO_3_ nanosheets were found to have predominantly exposed (002) crystal facets, accounting for over 90% of the total facet area. Compared to WO_3_ nanostructures with other morphologies synthesized without surfactant, the 2D nanosheets exhibited remarkably enhanced gas sensing performance towards xylene. Specifically, the 2D WO_3_ nanosheets showed a xylene sensing response of 57.5 at 300 °C, which was nearly 2 times higher than cubic WO_3_ nanoparticles. The significantly improved gas sensing and photocatalytic performance is attributed to two main factors - the high percentage of exposed (002) facets and the high specific surface area of 121 m^2^/g resulting from the ultrathin 2D morphology. Previous studies have shown that (002) crystal facets of monoclinic WO_3_ have higher surface energy and improved charge carrier separation compared to other facets like (020) and (200) [[45], [46], [47], [48]], leading to higher intrinsic reactivity. The 2D nanosheets maximize the exposure of reactive (002) facets, providing abundant active sites for interaction with target gas molecules during sensing.

**Fig. 2:**
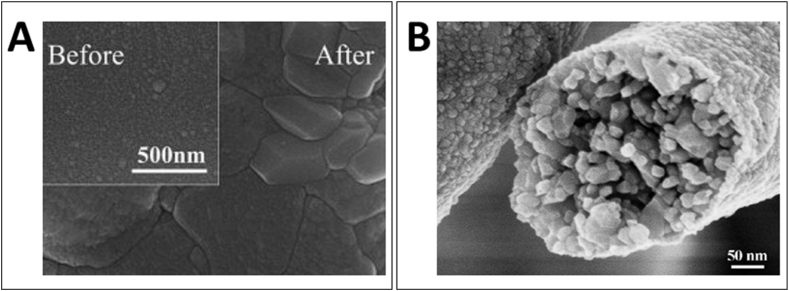
SEM images of (A) sputtered WO_3_ film before and after annealing (B) WO_3_ nanofibers after calcinations [50,51]. Permission obtained from ELSEVIER.

Smaller grains and porous morphologies provide higher surface area for gas interaction [49]. For example, Zeng et al. [50] demonstrated that porous WO_3_ gas sensors, prepared via anodic oxidation of sputtered metallic tungsten films, showcased a distinct advantage in terms of gas interaction due to their microstructure. The sensors were characterized by a coral-like porous crystalline structure with an exceptionally small grain size of approximately 9.3 nm, post-annealing (Fig. 2A). These attributes were pivotal in enhancing the sensor's performance, particularly for NO_2_ gas detection at a relatively low operating temperature of 150 °C, in stark contrast to the sputtered WO_3_ sensors. The larger specific surface area and reduced grain size of the porous sensors resulted in a markedly higher response to NO_2_ gas, underpinned by better response-recovery characteristics and a lower optimal operating temperature. The porous structure not only conferred a greater specific surface area but also offered more adsorption sites, thus facilitating an increase in gas molecule adsorption which, in turn, led to a more significant change in resistance upon exposure to NO_2_. Furthermore, the reduced grain size within the porous structure significantly contributed to the heightened sensor response. This correlation between smaller grain size and improved sensor sensitivity was corroborated by the grain size effects on gas sensitivity. A similar result can be found in work published by Wei et al. [51]. They fabricated porous WO_3_ nanofibers through an electrospinning method followed by calcination (Fig. 2B). The resultant nanofibers were predominantly comprised of minute grains with diameters averaging around 12 nm, a critical structural aspect influenced significantly by the calcination temperature. This fine granularity was pivotal as it led to a marked increase in surface area, verified by BET analysis to be an impressive 107.6 m^2^/g, thus substantiating the premise that smaller grains enhance surface area. These porous structures, endowed with a plethora of voids between grains, presented an advantageous morphology for gas interaction, as evidenced by the exceptional sensitivity to acetone. At an optimal temperature of 270 °C, the sensors demonstrated a profound response and selectivity toward acetone vapor, attributable to the high surface area facilitating abundant gas adsorption sites. Furthermore, the sensors exhibited rapid response and recovery times, which were ascribed to the efficient pathways for gas transport provided by the porous morphology. The BET analysis revealed a large textural porosity with a significant hysteresis loop in the nitrogen adsorption-desorption isotherms and a pore volume concentrated around 9 nm, indicating an abundance of mesopores conducive to gas diffusion. These morphological features, such as the abundance of interconnected pores and the small grain size, were crucial for achieving a high surface area and, consequently, a higher interaction with gas molecules. The gas-sensing properties were markedly improved, with the nanofiber sensor displaying high selectivity and sensitivity, establishing an impressive detection limit down to 0.1 ppm for acetone. These findings were not only consistent with the initial hypothesis that smaller grains and porous morphologies provide higher surface areas for gas interaction but also demonstrated a direct correlation between these structural characteristics and the exceptional gas-sensing performance of the WO_3_ nanofibers.

Oxygen vacancies act as preferential sites for gas molecule chemisorption [52]. Wang et al. [53] elucidated the role of oxygen vacancies in gas molecule chemisorption, particularly for ammonia, the research disclosed that these vacancies substantially enhance the sensitivity and selectivity of WO_3_ gas sensors. This sensitivity is attributed to the improved electron transportation efficiency at the material's surface, which facilitates a stronger and more rapid interaction with the gas molecules. The study's empirical findings were drawn from synthesized WO_3_ microspheres, which were specifically engineered to include oxygen vacancies. These microspheres demonstrated an outstanding ammonia sensing performance, exhibiting a response intensity 2.6 times higher than that of commercial WO_3_, absent of such vacancies. This superior performance was not attributed to an increased surface area, as both the as-prepared and commercial WO_3_ displayed similar surface areas, but rather to the strategic incorporation of oxygen vacancies within the WO_3_ structure. The presence of oxygen vacancies was rigorously verified through various advanced analytical methods. UV–visible–NIR DRS confirmed the optical signatures characteristic of oxygen vacancies, while XPS provided evidence of the W^5+^ states, indicative of the oxygen vacancies. Raman spectroscopy further substantiated these findings, revealing spectral features consistent with the structural disruption caused by these vacancies. EIS analyses underscored the reduced impedance of the as-prepared WO_3_, affirming the role of oxygen vacancies in facilitating charge transfer processes, essential for the heightened sensor response. In practical terms, the as-prepared WO_3_ microspheres not only demonstrated a heightened sensitivity to ammonia but also showcased remarkable stability and repeatability in response, vital for real-world sensing applications.

In the study conducted by Wang et al. [54], Co_3_O_4_-functionalized WO_3_ hollow microspheres were synthesized to improve the sensing properties for the detection of toluene. This research provided empirical evidence that oxygen vacancies serve as preferential sites for gas molecule chemisorption, particularly relevant to toluene. The investigation into the gas-sensing mechanism revealed that the presence of Co_3_O_4_ nanoparticles significantly enhanced the sensing performance compared to pure WO_3_ microspheres due to several factors. The introduction of Co_3_O_4_ was found to increase the amount of chemisorbed oxygen species and oxygen vacancies. The study reported that the response value of the composite to 100 ppm toluene reached 55.8, which is nearly three times higher than that of the pure WO_3_ hollow microspheres, demonstrating a direct correlation between oxygen vacancies and improved chemisorption. The study's findings are supported by XPS results indicating an increased relative percentage of oxygen vacancies and chemisorbed oxygen in the Co_3_O_4_-functionalized WO_3_ composite compared to the pure WO_3_ hollow spheres. These results indicate a better adsorption capacity for ionized oxygen species in the functionalized composite. Additionally, the Co_3_O_4_ nanoparticles, laden with oxygen vacancies, played a dual role: they facilitated the increase in the amount of chemisorbed oxygen on the surface and captured electrons from WO_3_, leading to a significant increase in resistance—a measure directly related to the sensor's response.

The underlying gas sensing mechanism in WO_3_ relies on changes in surface electron concentration caused by redox reactions with target gas molecules. Oxygen ionosorption extracts electrons from WO_3_, while interaction with reducing gases injects electrons back into the conduction band. This modulates the surface electron depletion layer and resistance. Factors like operating temperature, crystal structure, exposed facets, porosity and noble metal doping significantly impact the gas adsorption and reaction kinetics governing the sensor response and sensitivity. Advances in nanostructure morphology control and hybrid nanocomposites provide opportunities to further tune the WO_3_ gas sensing performance.

## 3 Synthesis techniques for WO_3_ gas sensing materials

Various synthesis approaches have been employed to obtain WO_3_ nanostructures for gas sensing applications. The fabrication technique plays a key role in controlling the morphology, exposed facets, crystallite size, porosity and specific surface area. These structural parameters significantly impact the gas accessibility, adsorption-desorption kinetics and sensitivity. The synthesis methods can be broadly classified into wet chemical routes and physical vapor deposition.

### 3.1 Wet chemical methods

#### 3.1.1 Hydrothermal/solvothermal synthesis

**Fig. 3:**
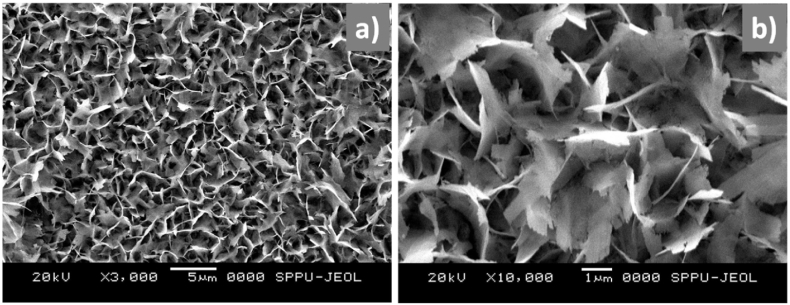
SEM micrographs of WO_3_ nanoflakes thin film a) lower magnified image; b) higher magnified image [55]. Permission obtained from ELSEVIER.

Hydrothermal technique involves heating the precursors in an aqueous medium in a sealed autoclave at temperatures above the boiling point of water. It enables crystallization and growth of nanostructures under high temperature and autogenous pressure. The reaction time, temperature, solvent composition and use of surfactants or structure-directing agents allow morphology control of the resulting WO_3_. For example, Kolhe et al. [55] synthesized WO_3_ nanoflake thin films via a hydrothermal route, primarily for gas sensing applications (Fig. 3a and b), with a particular emphasis on NH_3_ detection. The hydrothermal method was chosen for its ability to control morphology and size, which are critical for the sensing properties of materials. The WO_3_ thin films, developed on an FTO substrate with a monoclinic structure. The gas sensing studies revealed that the WO_3_ nanoflakes exhibited a superior sensor response, particularly to NH_3_, demonstrating higher sensitivity compared to other gases like H_2_S and CO. This response to NH_3_ was found to be around 73% at an optimal operating temperature of 150 °C with quick response (28 s) and recovery times (68 s). The study highlighted the benefits of the hydrothermal route in achieving nanoflakes morphology, which provides a large surface area with abundant active sites for gas molecule interaction, leading to enhanced gas sensing performance. Moreover, the interconnected nanoflakes facilitated charge transportation, which is beneficial for sensor sensitivity. In another work [56], researchers focused on the hydrothermal synthesis of monodisperse h-WO_3_ nanowires and the examination of their performance as gas sensors in thin film form. The nanowires, synthesized through the acidification of sodium tungstate by potassium and sodium sulfate, exhibited high crystallinity and uniformity, crucial for sensitive gas detection. The primary purpose of using the hydrothermal route was to create h-WO_3_ nanowires with good dispersity and exposure to crystal facets beneficial for gas sensing. The method proved to be cost-effective and simple, yielding nanowires with properties that enhance gas sensor function. The performance of these nanowires in gas sensors was specifically tested against ethanol and formaldehyde, achieving high responsiveness at low concentrations (10 ppm), with exceptional response and recovery times as short as 4–8 s. Such quick response times are notable compared to existing sensors.

**Fig. 4:**
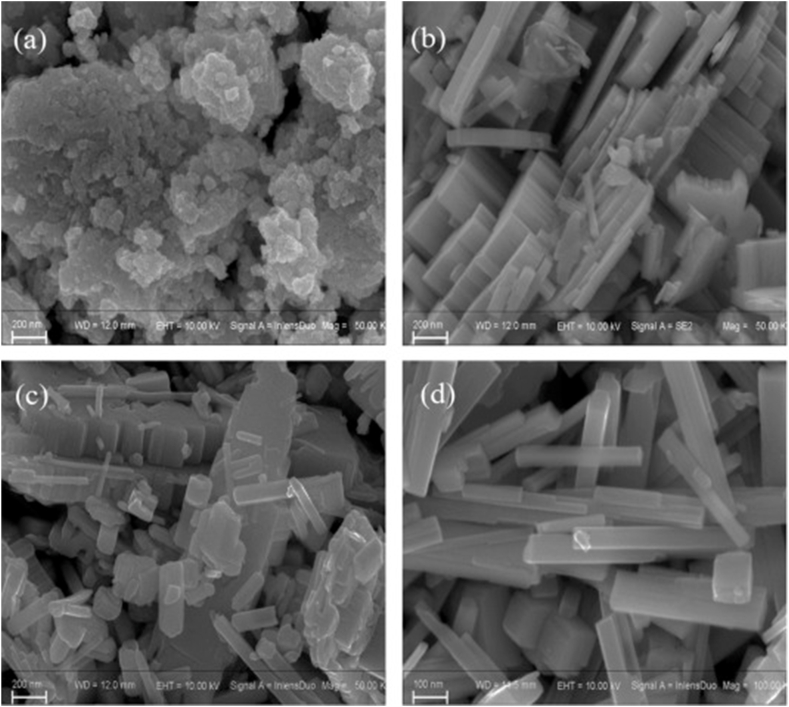
SEM images of the as-prepared samples after heating treatment synthesized at different reaction temperature: (a) 170 °C, (b) 180 °C, (c) 190 °C, (d) 200 °C [57]. Permission obtained from ELSEVIER.

Solvothermal synthesis is similar but uses non-aqueous solvents. In a previous study [57], WO_3_ nanocrystals were solvothermally synthesized, demonstrating that the reaction temperature critically influenced their morphology and, consequently, their gas-sensing properties. Specifically, these nanocrystals were prepared by dissolving tungsten hexachloride in ethylene glycol and water, then undergoing a solvothermal reaction in a Teflon-lined autoclave at various temperatures ranging from 170 °C to 200 °C. The resulting morphologies ranged from homogeneous nanoparticles at 170 °C to nanorods at 200 °C (Fig. 4a-d), with a direct correlation between the increased reaction temperature and the evolution of these structures. The sensors, created from these WO_3_ nanocrystals, exhibited distinct NO_2_-sensing characteristics that varied with the reaction temperatures. Notably, sensors synthesized at 170 °C and 180 °C demonstrated an unusual p-type semiconducting behavior at temperatures below 38 °C and 55 °C, respectively. This phenomenon was attributed to an inversion layer formation on the n-type WO_3_ nanocrystals, where oxygen adsorption caused a transformation in the surface conduction type from n-type to p-type. The study underscored the importance of controlling the solvothermal reaction temperature to tailor the morphology and enhance the functionality of WO_3_ nanocrystals for sensitive NO_2_ detection at low temperatures. A similar approach has also been used for synthesis of monoclinic WO_3_ quantum dots [58]. The synthesized nanocrystals maintained the stable bulk monoclinic phase even at a nanoscale size, averaging 4 nm. These nanocrystals demonstrated remarkable gas-sensing capabilities for both reducing (ethanol) and oxidizing (nitrogen dioxide) gases at low concentrations, exhibiting responses over two to three orders of magnitude at operating temperatures of 100 °C and 200 °C, respectively. The enhanced sensing performance was attributed to the reduced surfaces of the nanocrystals, which facilitated nitrogen dioxide adsorption and oxygen ionosorption, thereby improving ethanol decomposition kinetics. The solvothermal synthesis approach allowed for easy processing of gas-sensing devices without phase transition up to at least 500 °C and provided a means to control the growth and properties of the WO_3_ quantum dots, which is critical for the development of high-performance gas sensors.

Microwave-assisted solvothermal/hydrothermal technique allows rapid heating by microwave dielectric heating effects. For example, a combination of hemispherical WO_3_ and graphene, is synthesized using a microwave-assisted hydrothermal method [59]. The study demonstrated that the inclusion of graphene significantly influences the crystal structure evolution of the WO_3_, transitioning it from nanoparticles to a hemispherical structure. This structural transformation is crucial as it substantially enhances the gas-sensing abilities of the composite, particularly for amine gases like triethylamine, even at room temperature. The hollow, hemispherical structure, facilitated by the presence of graphene, offers more surface reaction sites and effectively modulates the electron density across the entire volume of the composite. This morphological change can be attributed to two key factors - firstly, graphene's high surface area and electron mobility allows deposited WO_3_ units to migrate and reconfigure on its surface. Secondly, strong interfacial bonding distributes strain between the graphene and WO_3_, leading to the curved hemispherical shapes which provide more uniform gas access to the entire composite volume. This results in a markedly improved gas-sensing response. The study's findings reveal that the microwave-assisted method is not only efficient in synthesizing this nanocomposite but also plays a vital role in achieving its exceptional gas-sensing capabilities. Wang et al. [60] synthesized flower-like WO_3_ architectures through a simple, surfactant-free microwave-assisted solvothermal process, followed by calcination. The purpose of employing microwave synthesis is central to the work. This method offers a rapid, efficient, and environmentally friendly alternative for creating complex nanostructures. They emphasized the microwave method's ability to produce high-quality nanostructures with desirable properties for gas sensing applications. The resultant WO_3_ nanostructures, with their unique flower-like morphology, showed promising characteristics for low-level NO_2_ detection and fast response to volatile organic compounds like acetone.

#### 3.1.2 Sol-gel process

The sol-gel process involves hydrolysis and condensation of molecular precursors to form a colloidal suspension (sol) which converts to a solid porous network (gel) after drying. Common precursors for WO_3_ synthesis are tungstates like ammonium paratungstate (APT) or tungstic acid which undergo polycondensation reactions. Surfactants are used to obtain porous structures.

**Fig. 5:**
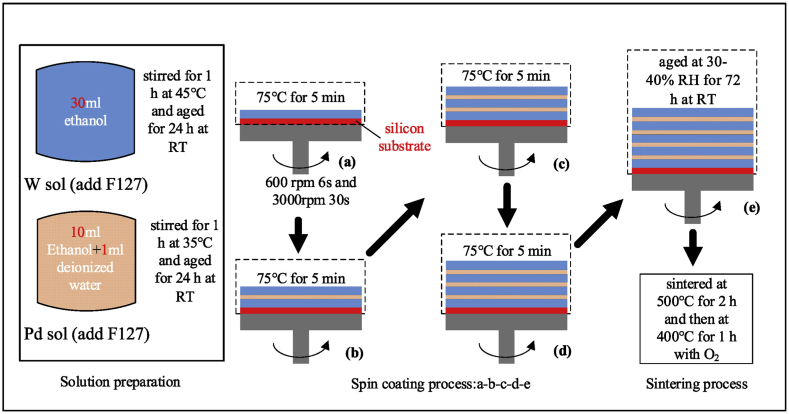
Schematic illustration for the preparation of multilayer porous Pd-WO_3_ composite films [61]. Permission obtained from ELSEVIER.

Sol-gel enables excellent control over morphology and porosity. Han et al. [61] focused on the fabrication of multilayer porous Pd-WO_3_ composite thin films using a sol-gel method, aimed at enhancing hydrogen sensing capabilities (Fig. 5). Key findings included that the optimal molar ratio of Pd:W was 1%, which yielded the most effective hydrogen sensing performance. The films exhibited a significant improvement in hydrogen sensitivity—approximately 346.5 times greater than pure WO_3_ films. This enhanced sensitivity was attributed to the films' porous structure, which provided additional active sites for hydrogen detection. The 1 mol% porous Pd-WO_3_ composite films demonstrated a rapid response time of just 7 s and maintained stable sensing performance. Moreover, these films showed notable selectivity for hydrogen, with about 20 times higher sensitivity to hydrogen than to other gases like CO and CH_4_. In another work [62], sodium tungstate dehydrate and hydrochloric acid were used to prepare WO_3_ nanostructures. The pH of the solution was varied (1, 1.5, 2), and the effects were studied both on as-synthesized and calcined (at 500 °C) nanoparticles. The research found that the pH significantly influenced the nanoparticles' morphology, crystallinity, chemical bonds, and optical properties. An increase in pH led to a change in the crystal phase from orthorhombic to hexagonal in as-synthesized samples and further to monoclinic upon calcination. As the pH increased, the nanoparticle size also grew in as-synthesized samples but reduced upon calcination due to the evaporation of structural water and hydrate groups. They found that WO_3_ thin films calcined at 500 °C exhibited the highest sensitivity for CO gas sensing.

#### 3.1.3 Precipitation

This involves inducing precipitation of solid WO_3_ particles by changing the solubility of the dissolved tungsten precursor. Precipitating agents like HCl, HNO_3_, (NH_4_)_2_SO_4_ are added to metal salt solutions to generate the precipitate which is washed, dried and calcined.

**Fig. 6:**
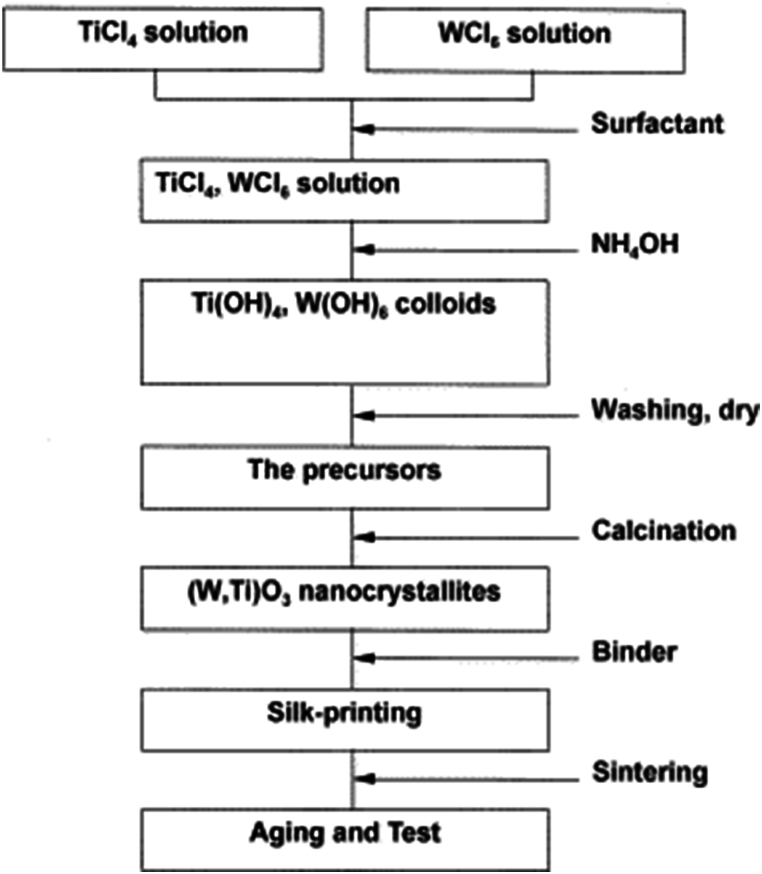
Schematic process for a TiO_2_–WO_3_ based NO_2_ sensor fabrication [64]. Permission obtained from ELSEVIER.

In a study exploring the fabrication of WO_3_/multi-walled carbon nanotubes (MWCNT) hybrid materials for gas sensing applications [63], researchers utilized an acid precipitation method. This approach involved combining ammonium tungstate *para*-pentahydrate with MWCNTs, followed by calcination at 300–600 °C. The resulting hybrid materials exhibited high surface area and mesoporosity, crucial for effective gas sensing. The researchers observed that the specific surface area of the hybrid materials decreased with higher calcination temperatures, due to particle agglomeration. However, the optimal calcination temperature was determined to be 400 °C, balancing the need for high surface area with the avoidance of MWCNT combustion. This temperature allowed for a uniform dispersion of WO_3_ on the MWCNT surface. Lee et al. [64] explored the TiO_2_-adding method in NO_2_-sensing characteristics and surface properties of two TiO_2_-WO_3_ nanocrystallite sensors prepared by coprecipitation and precipitation methods (Fig. 6). It revealed that the coprecipitated nanocrystallites of the TiO_2_-WO_3_ sensor exhibited finer particles, smaller agglomerates, and larger surface area than those prepared by the precipitation method. This distinction in microstructure translated into improved sensitivity and sorption properties for the coprecipitated materials. The coprecipitated materials demonstrated a significant enhancement in gas-sensing performance. For instance, the sensitivity, defined as the ratio of electrical resistance in a gas environment to that in clean air, was around 100 for 30 ppm NO_2_ at 340 °C, making it suitable for use in facility combustion furnaces. The coprecipitated sensor showed a high sensitivity of about 1650 at an operating temperature of 180 °C, which decreased at higher temperatures due to the fundamental resistance-temperature relationship of the semiconductor material. Kabcum et al. [65] focused on developing ultra-responsive hydrogen sensors using Pd-loaded WO_3_ nanorods via a modified precipitation method, utilizing ethylene glycol as a dispersing agent and then impregnated with Pd nanoparticles. The Pd-loaded WO_3_ nanostructures were composed of 5–20 nm spherical or oval PdO nanoparticles dispersed on the surface of polycrystalline WO_3_ nanorods. These were applied to create sensing films on alumina substrates with interdigitated gold electrodes. The research demonstrated that the sensors operated optimally at a low temperature range (25–350 °C), with varying Pd loading levels from 0 to 2 wt%. Notably, a 1 wt% Pd-loaded WO_3_ sensing film showed the highest response of approximately 3.14 × 10^6^, with a rapid response time of 1.8 s–3 vol% H_2_ at an optimal operating temperature of 150 °C.

#### 3.1.4 Combustion synthesis

Combustion synthesis uses a self-propagating exothermic redox reaction between the precursor and a fuel to produce WO_3_ nanopowders.

**Fig. 7:**
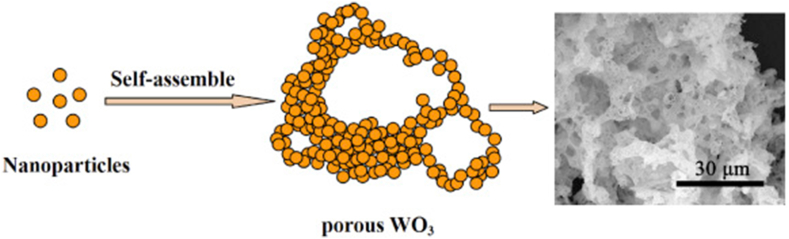
A possible growth mechanism of porous WO_3_ [67]. Permission obtained from ELSEVIER.

In a groundbreaking study, Morales et al. [66] demonstrated the efficacy of combustion synthesis in producing nanocrystalline WO_3_. It facilitated the creation of nanosized WO_3_ particles by using simple fuels like glycine, urea, or thiourea in a combustion process. The resultant WO_3_ showed enhanced optical characteristics, with the ability to shift its response towards the visible spectrum. Moreover, the synthesized WO_3_ demonstrated superior surface properties. For instance, it exhibited significantly improved organic dye uptake compared to commercial samples. The study's success in efficiently producing WO_3_ with tailored optical and surface properties using combustion synthesis marks a significant advancement in the field of material science, particularly for renewable energy applications. Dong et al. [67] focused on the synthesis of hierarchically porous WO_3_ using a combustion synthesis method. This method involved dissolving tungsten powder in hydrogen peroxide, followed by a reaction with a combined fuel of glycine and hydrazine hydrate. During the combustion synthesis process, WO_3_ was formed from the decomposition of a tungsten-based complex. This formation process was driven by the need to reduce surface energy, which led to the aggregation of a large number of WO_3_ nanoparticles (Fig. 7). As these nanoparticles aggregated, they allowed gases such as CO_2_, N_2_, and water vapor to pass through, contributing to the development of a hierarchically porous structure. The main results revealed that the porous WO_3_ sensor displayed remarkable gas sensing characteristics. It showed a high gas response, rapid response and recovery times, good reproducibility, and excellent selectivity towards acetone. The study highlighted that the combustion synthesis method was a simple, eco-friendly, and cost-effective approach for producing metal oxides with superior gas sensing properties. The hierarchical porous structure created through this method played a crucial role in enhancing the sensor's performance by offering an increased number of reactive sites for the gas molecules.

#### 3.1.5 Flame spray pyrolysis

**Fig. 8:**
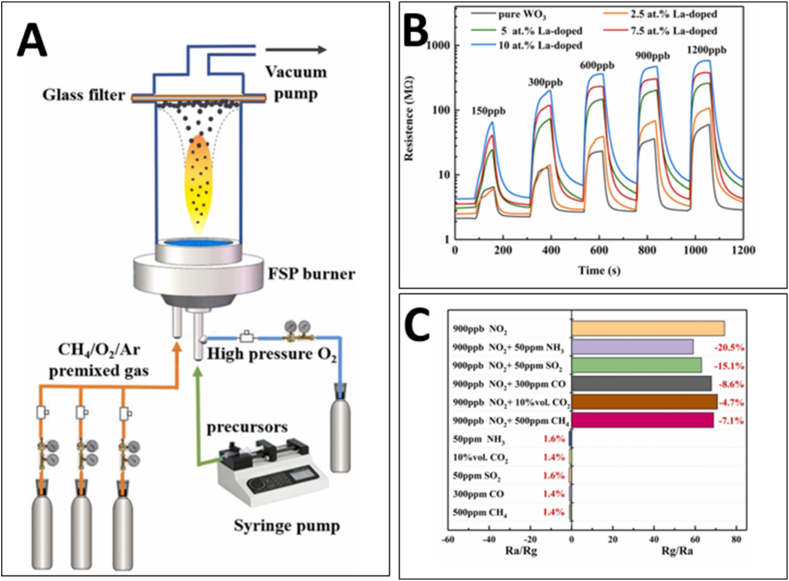
(A) The experimental setup of flame spray pyrolysis system. (B) Dynamical response-recovery curves of the WO_3_ sensing materials with different La-doping ratios. (C) Cross-sensitivity of the 7.5 at% La-doped WO_3_ to various interference gases [68]. Permission obtained from ELSEVIER.

In flame spray pyrolysis, the solution containing dissolved precursors is sprayed into a flame through a nozzle, resulting in ultrafine nanoparticle powders. Gases like methane are used as fuel while O_2_ provides the high temperature oxidation zone. This enables large scale synthesis of pure and doped WO_3_ nanopowders as well as direct deposition as thin films. For instance, Zhang et al. [68] synthesized lanthanum-doped WO_3_ nanoparticles using flame spray pyrolysis and demonstrated their superior performance as NO_2_ sensors (Fig. 8A shows the experimental setup of flame spray pyrolysis system). This method enabled the homogenous dispersion of doped lanthanum atoms on WO_3_ particles in the form of La_2_O_3_, which was pivotal in enhancing the nanoparticles' sensing capabilities. The team discovered that the optimal doping ratio of lanthanum was 7.5 at%, which yielded the highest sensing response of 74.2 towards 900 ppb NO_2_ at 125 °C, with response and recovery times of 23 and 35 s, respectively (Fig. 8B). The study revealed that the enhanced sensing performance resulted from the enriched vacancy oxygen and additional absorption sites provided by the La doping, as well as the creation of p-n heterojunctions that facilitated more effective electron interaction with NO_2_. The 7.5 at% La-doped WO_3_ sensor also exhibited exceptional anti-interference performance against gases like NH_3_, SO_2_, CO, CO_2_, and CH_4_, and showed minimal disturbance in the presence of ppm-level NH_3_ and SO_2_ coexisting with NO_2_ (Fig. 8C). Furthermore, the sensor demonstrated good resistance to humidity fluctuations and maintained stability over time, proving its potential for practical applications. These findings marked a significant advancement in the field of gas sensing, highlighting the effectiveness of flame spray pyrolysis as a synthesis method for creating high-performance NO_2_ sensors.

#### 3.1.6 Chemical bath deposition (CBD)

In CBD, the substrate is immersed in the precursor solution along with complexing agents. The reactants undergo slow release and controlled precipitation on the substrate, resulting in growth of adherent WO_3_ films. CBD enables good control over film thickness and morphology at low temperatures. Wang et al. [69] synthesized WO_3_ porous nanosheet arrays (PNAs) using the CBD method. This approach facilitated in-situ growth of nanosheet-assembled spheres, which upon annealing at 400 °C, transformed into porous nanosheets due to the removal of crystalline water. These PNAs, with a thickness of approximately 20 nm, demonstrated exceptional gas sensing performance towards NO_2_. A notable feature of the PNAs was their enhanced low-temperature gas sensing capabilities. At an operating temperature of just 100 °C, the WO_3_ PNAs achieved a high response of 460 towards 10 ppm NO_2_. This performance significantly surpassed that of a thicker WO_3_ layer, highlighting the efficiency of the PNAs' structure in gas detection. The superior sensing characteristics were attributed to the high degree of surface participation in the reaction with the gas, facilitated by the porous structure of the nanosheets. The study also explored the growth mechanism of the precursor nanosheets and the phase transformation from tungstite to monoclinic WO_3_. It was observed that the WO_3_ PNAs exhibited high selectivity for NO_2_ over other poisonous gases like SO_2_, H_2_S, CO, and NH_3_ at the same operating temperature. The temperature-dependent gas response was interpreted as a result of competitive adsorption of oxygen and NO_2_ at low temperatures and their desorption at high temperatures. Yao et al. [70] recently investigated the detail of WO_3_ growth under CBD condition. They developed a method for synthesizing WO_3_ films on a fluorine-doped tin oxide (FTO) substrate using a two-step chemical bath deposition-annealing process. The key aspect of this process was the regulation of film growth and thickness by controlling the amounts of reactants, particularly oxalic acid dihydrate (H_2_C_2_O_4_⋅2H_2_O), which acted as a growth controller. This method allowed for the creation of WO_3_ films with varied thicknesses, influencing their morphological and optical properties. The study proposed a three-step growth mechanism for the H_2_WO_4_ layer on the FTO substrate, which involved the formation of separate nanosheets, growth of secondary nanosheets forming clusters, and the eventual connection of these clusters to form a continuous film. This mechanism was significant in understanding the growth processes of films constructed from nanosheets.

### 3.2 Physical vapor deposition

#### 3.2.1 Thermal evaporation

Thermal evaporation relies on physical vaporization of the source material by heating under vacuum and its deposition on a cooler substrate to grow thin films. The vapor pressure, deposition rate, substrate temperature and annealing conditions allow tuning of WO_3_ film properties. Ponzoni et al. [71] developed nanostructured WO_3_ films using a modified thermal evaporation technique. This method involved sublimating metallic tungsten wire followed by oxidation in a low vacuum and reactive atmosphere, with substrates heated at high temperatures (600 °C). The resulting films displayed high surface roughness and a large effective area, making them well-suited for gas-sensing applications. SEM and AFM analyses revealed that these films consisted of nanometric-sized agglomerates. The performance of these nanostructured WO_3_ films in gas-sensing was particularly noteworthy. The films showed excellent sensitivity, especially at a lower working temperature of 100 °C. They demonstrated high responses to sub-ppm concentrations of NO_2_, outperforming responses to other gases like NH_3_ and CO. This result was significant compared to sensors based on sputtered thin films, where the thermally evaporated films exhibited improved performance. At 100 °C, the sensors based on these films showed a strong selectivity towards NO_2_ against NH_3_ and CO, with the ability to detect NO_2_ concentrations as low as under 100 ppb. Additionally, the study found that the sensing performance for NO_2_ was enhanced with decreasing humidity. The response times of the sensors also decreased with lower temperatures, reaching around 160 s at 100 °C, which was comparable to the chamber filling time. Na et al. [72] investigated the surface morphology and sensing properties of WO_3_ and NiO-WO_3_ thin films prepared via the thermal evaporation method. The films were deposited on Al_2_O_3_–Si substrates and annealed at 500 °C for 30 min. The study revealed that WO_3_ thin films, when increased in thickness, developed cracks between polycrystalline grains, leading to degraded sensing characteristics. However, an optimal deposition of NiO on WO_3_ films significantly improved their sensitivity by inhibiting grain growth. This inhibition was effective only up to a certain thickness of WO_3_ and NiO content, beyond which the grain growth control was not effective. Moreover, the deposition sequence of NiO and WO_3_ played a crucial role in controlling grain growth. The most effective method to suppress grain growth was found to be the deposition of NiO above the WO_3_ films. These findings indicate that the surface morphology of WO_3_ and NiO-WO_3_ thin films, crucial for their sensing properties, can be effectively controlled through thermal evaporation and the strategic deposition of NiO.

#### 3.2.2 Sputter deposition

In sputter deposition, plasma is created using an inert gas like Ar which accelerates ions to bombard the target. This results in ejection of target atoms that condense as a thin film on the substrate. Magnetron sputtering uses magnetic fields to enhance plasma density and deposition rate. Both direct current (DC) and radio frequency (RF) sputtering have been employed for WO_3_ deposition.

**Fig. 9:**
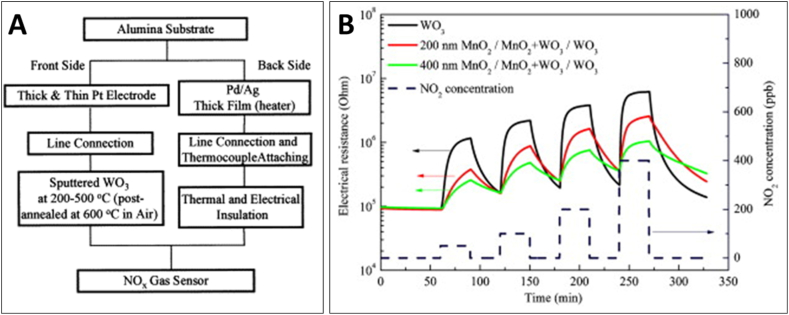
(A) Schematic diagram of NOx sensor fabrication using DC reactive sputtering [73]. (B) Three RF sputtering fabricated sensors toward 50, 100, 200 and 400 ppb NO_2_ at 200 °C [74]. Permission obtained from ELSEVIER.

Kim et al. [73] reported on the use of DC reactive sputtering for the fabrication of WO_3_ thin films, with an emphasis on their application as NO gas sensors. The key findings were centered around the improvement of the sensor's performance through the adjustment of deposition temperatures and post-annealing processes. Initially, the WO_3_ thin films were deposited on an alumina substrate at temperatures ranging from 200 °C to 500 °C, followed by a post-annealing step at 600 °C. This process was found to significantly enhance the crystallinity of the films (Fig. 9A). It was observed that films deposited at lower temperatures exhibited lower crystallinity, impacting their sensitivity as gas sensors. The study demonstrated that the crystallinity of the WO_3_ thin films was crucial for effective gas sensing. The performance of the sensors was evaluated based on their sensitivity to NO gas at concentrations of 1–5 ppm. The sensitivity of the as-deposited thin films varied between 4 and 10 for a 5 ppm NO test gas at a measuring temperature of 200 °C. However, post-annealing at 600 °C dramatically increased this sensitivity to values ranging between 70 and 180 under the same test conditions. This indicated that a post-annealing process at a minimum of 600 °C was necessary for optimal sensor performance.

In another study [74], WO_3_ sensors equipped with MnO_2_ filters were developed for precise NO_2_ detection, employing RF sputtering for film deposition. The combination of WO_3_ active layers, WO_3_ + MnO_2_ insulating layers, and MnO_2_ filters was effectively deposited using RF sputtering, followed by an annealing treatment at 450 °C for 24 h. This method was chosen for its ability to create thin films with precise control over thickness and composition, crucial for sensor functionality. The main achievement of this study was the successful reduction of O_3_ interference in NO_2_ detection. The sensors maintained high sensitivity to NO_2_ concentrations ranging from 50 to 400 ppb (Fig. 9B), across a temperature range of 150–250 °C. Notably, the MnO_2_ filters significantly reduced the response to O_3_ (only 1–3% compared to sensors without the filter), effectively addressing a common challenge in gas sensor technology—selectivity.

#### 3.2.3 Pulsed laser deposition (PLD)

**Fig. 10:**
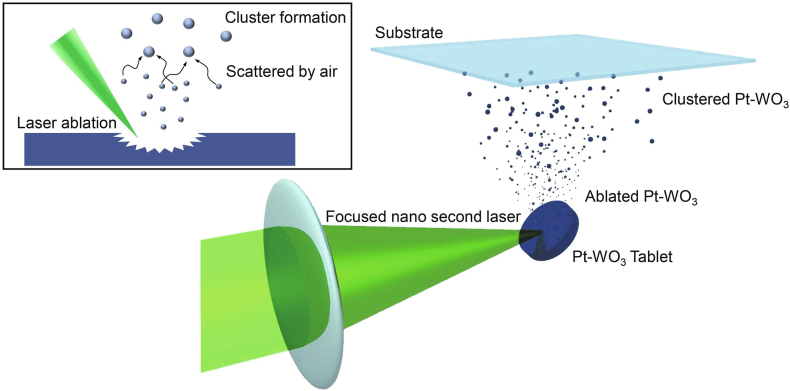
Schematic illustration of the PLD under atmospheric pressure conditions [75]. Permission obtained from ELSEVIER.

In PLD, short and intense laser pulses ablate the target leading to plasma plume formation containing ejected material which condenses as a thin film on the substrate. This enables stoichiometric transfer of target composition. The film properties can be tuned by laser parameters, ambient gas pressure and substrate temperature. PLD has been used for fabricating a hydrogen gas sensing using Pt-WO₃ nano-/micro-powder films [75]. This method, performed under atmospheric conditions, employed an all-optical, non-contact technique to measure electromagnetic radiation transmittance in the near-IR 1.3 μm telecommunications frequency band, allowing for rapid detection of low-concentration hydrogen gas. The PLD method under atmospheric conditions proved advantageous for creating materials with a rough and porous topology, thereby enhancing the gas molecule interaction due to an increased surface area (Fig. 10). This was a critical factor in improving both the response rate and sensitivity of the sensors. The study's results showed that the hydrogen response of the Pt-WO_3_ composites was highly dependent on the Pt content. Increasing the Pt fraction in the composite led to a notable decrease in both the hydrogen uptake and release time-constants. Specifically, composites with a 50:1 WO_3_-to-Pt ratio demonstrated particularly swift hydrogen uptake times between 20 and 23 s and hydrogen release durations in the 27–37 s range. This performance was remarkable given the low concentrations of hydrogen (below 4% by volume) involved in the study. The hydrogen detection capability of these composites was quantified, revealing a close-to-linear dependence of extinction loss on hydrogen concentration. The detection limit of different samples, based on the Pt mass fraction in the composite material, showed that the limit of H₂ detection was estimated at around 10 ppm, with a response time of approximately 20 s at low hydrogen concentrations.

## 4 WO_3_ nanostructures for enhanced gas sensing

Engineering WO_3_ at the nanoscale offers unique opportunities to tune its gas sensing properties. Key parameters that determine the sensor response and kinetics include the specific surface area, porosity, crystal facets exposure and oxygen vacancies. These can be optimized by synthesizing different WO_3_ nanostructures using techniques discussed in the previous section.

### 4.1 0D nanostructures

**Fig. 11:**
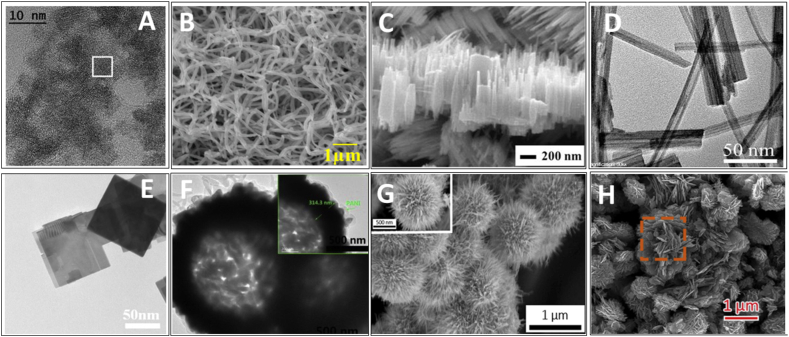
(A) HRTEM image of WO_3_ quantum dots [58]. SEM image of (B) WO_3_ nanofibers [79] and (C) WO_3_ nanorods [80]. HRTEM image of (D) WO_3_ nanotubes [81], (E) nanosheets [82] and (F) PAWHs [83]. SEM image of (G) urchins-like WO_3_ [84] and (H) flower-like WO_3_ [85]. Permission obtained from ELSEVIER and MDPI.

0D nanostructures refer to nanoparticles, quantum dots, nanocubes and other ultra-small morphologies. While they exhibit high electrical resistance due to lack of interconnectivity, strategies like decoration onto graphene have been used to improve conductance [76,77]. A study conducted by Qin et al. [77] presented a novel approach to synthesize graphene-wrapped WO_3_ nanoparticles. The researchers developed a three-step synthesis process, which began with the creation of an SrWO_4_/graphene oxide precursor through homogeneous precipitation. This precursor was then converted into WO_3_/GO hybrids using acidification, followed by a reduction to WO_3_/graphene nanocomposites via UV-assisted photoreduction in water. This method is particularly notable for its room-temperature operation and avoidance of typical alcoholic solvents. The study's findings showed that the WO_3_ nanoparticles, with a size of 50–200 nm, were effectively anchored on graphene sheets, serving as spacers to keep neighboring sheets separated. These nanocomposites demonstrated significantly enhanced electrical conductivity compared to WO_3_/GO hybrids, leading to improved gas sensing properties, especially towards alcohol vapors. Epifani et al. [58] synthesized monoclinic WO_3_ quantum dots through solvothermal processing, using W-chloroalkoxide solutions in oleic acid at 250 °C. These quantum dots, averaging 4 nm in size (Fig. 11A), retained the bulk monoclinic crystallographic phase even in their nanosized form. It was found that the nanocrystals had a core of monoclinic WO_3_ with a surface covered by W(V) species, which slowly oxidized in room conditions. The WO_3_ nanocrystals demonstrated exceptional capabilities in gas sensing. They were processed into gas-sensing devices without undergoing any phase transition up to 500 °C. These devices showed notable sensitivity to both oxidizing (NO_2_) and reducing (ethanol) gases at concentrations ranging from 1 to 5 ppm for NO_2_ and 100–500 ppm for ethanol. Remarkably, the devices operated at low temperatures of 100 and 200 °C for NO_2_ and ethanol, respectively. The enhanced sensing performance was attributed to reduced surfaces and increased oxygen ionosorption, leading to improved NO_2_ adsorption and accelerated ethanol decomposition kinetics. Yu et al. [78] also synthesized WO_3_ quantum dots for H_2_S gas detection. The WO_3_ quantum dots were created through a colloidal synthesis process and employed in sensor devices fabricated at room temperature, avoiding the need for high-temperature sintering. The synthesized WO_3_ quantum dots-based sensors demonstrated a maximum response of 57 towards 50 ppm of H_2_S at an optimal temperature of 80 °C, with a response time of 47 s and a recovery time of 126 s. The sensors displayed excellent reversibility and minimal baseline drift, with a linear sensor response in the H2S concentration range of 5–25 ppm. The theoretical limit of detection was calculated to be as low as 56 ppb at 80 °C.

### 4.2 1D nanostructures

1D nanostructures include nanowires, nanofibers, nanotubes and nanorods which provide direct conduction pathways for electron transport. This enhances conductance and gas interaction through radially exposed surfaces. WO_3_ nanofibers can be simply synthesized using a novel one-step hydrothermal method [79]. These nanofibers, characterized by uniform size with diameters around 100 nm and lengths up to tens of micrometers (Fig. 11B), demonstrated exceptional gas sensing performance, especially towards ethanol. The optimal operating temperature for the sensor was identified as 350 °C, at which the response value to 100 ppm ethanol could reach as high as 62. The sensor also showed remarkable sensitivity to varying concentrations of ethanol, with response values ranging from 31.18 to 45.56 for concentrations between 10 ppm and 50 ppm. This high gas-sensing response was attributed to the unique morphology of the WO_3_ nanofibers, which featured a large specific surface area and abundant channels for gas diffusion and mass transport. These characteristics not only shortened the gas diffusion distance but also provided highly accessible open channels and active surfaces for the detected gas. WO_3_ nanorod arrays (Fig. 11C) can be synthesized via a substrate-free hydrothermal method [80]. The synthesis process involved the dissolution of sodium tungstate dehydrate and oxalic acid (OA) in a water-ethanol mixture, followed by heating in a Teflon-lined stainless-steel vessel. The study revealed that these WO_3_ nanorod arrays exhibited exceptional NH_3_ sensing capabilities. Specifically, the arrays demonstrated a remarkably high response of 8.3 at an NH_3_ concentration of 50 ppm and a temperature of 200 °C.

Ultrathin WO_3_ nanotubes can be synthesized through a hydrothermal method, employing K_2_SO_4_ and citric acid (CA) under controlled conditions [81]. The researchers engineered the morphology of these nanostructures, which exhibited dimensions such as a diameter of 10–15 nm and a wall thickness of 1–2 nm for nanotubes (Fig. 11D). The resulting WO_3_ nanotubes demonstrated exceptional gas sensing performance, particularly for detecting acetone and ethanol. Significantly, the WO_3_ nanotubes showed a superior sensing response (Ra/Rg) of 32 and 26 for acetone and ethanol, respectively. This enhanced sensitivity was attributed to the ultrathin wall structure, high surface area, and the presence of crystal defects and oxygen vacancies.

### 4.3 2D nanostructures

2D nanostructures like nanosheets, nanoplates and thin films maximize the material utilization by providing a high density of exposed surfaces available for gas interaction. Reducing the thickness to nanoscale minimizes bulk effects and enhances surface-to-volume ratio. WO_3_ nanosheets (Fig. 11E) prepared by microwave-assisted hydrothermal method showed excellent response for volatile organic compounds (VOCs) [82]. Three types of WO_3_ nanosheets were prepared using OA, CA, and tartaric acid (TA) as auxiliary agents. WO_3_-CA nanosheets exhibited significant sensitivity and superior performance in detecting formaldehyde, acetone, and various alkanes compared to WO_3_-OA and WO_3_-TA. This enhanced sensitivity was attributed to their abundant oxygen vacancies and a high surface charge migration rate, which provided more reaction sites for gas molecules. The study demonstrated that the gas sensitivity of WO_3_-CA was due to its specific structural properties, such as a higher content of (002) crystalline surface and more anion-adsorbed oxygen. In the study conducted by Liu et al. [86], WO_3_ nanoplates were synthesized using a hydrothermal method and used to create sensors for detecting acetone and ammonia gases at different operating temperatures. These nanoplates, characterized by their abundant surface chemisorbed oxygen species, exhibited significant gas sensing properties. Specifically, at a high operating temperature of 300 °C, the WO_3_ nanoplate-based sensor demonstrated a wide acetone detection range (1–500 ppm), with rapid response and recovery times (3 s and 7 s, respectively), good selectivity, and stability. At a lower operating temperature of 140 °C, the same sensor also showed promising performance in detecting ammonia gas. The differing sensing properties at varied temperatures were attributed to changes in active oxygen species on the WO_3_ surfaces and the differing bonding energies of acetone and ammonia molecules.

### 4.4 3D nanostructures

3D morphologies like hollow spheres, urchins, flowers and cubic assemblies provide an optimal combination of porosity, large surface area and good interconnectivity between the primary nanoscale building units. This results in excellent gas diffusion, accessibility and charge transport.

WO_3_ hollow nanospheres@polyaniline (PAWHs) prepared by template-assisted solvothermal method for room-temperature NH_3_ sensing [83]. The PAWHs10 hybrid (Fig. 11F), with a 10 mol% WO_3_ composition, exhibited the most remarkable performance, delivering a high response value of 25–100 ppm NH_3_ at 20 °C, which was approximately 4.2 times greater than that of unmodified PANI sensors. This sensor also achieved ppb-level detection limits (1.67–500 ppb), fast response/recovery rates (136 s/130 s), and excellent NH_3_ selectivity, outperforming other sensors in the study. The superior sensing performance was attributed to the unique hollow structure of WO_3_ and the formation of p-n heterojunctions between PANI and WO_3_ hollow spheres. These structural features not only increased the surface area available for gas adsorption and diffusion but also enhanced the sensitivity of the material to NH_3_.

Urchins-like WO_3_ assemblies fabricated by via a hydrothermal method demonstrated response of acetone concentrations ranging from 2 to 5000 ppm at 200 °C [84]. Specifically, the response to 100 ppm acetone reached 29.7, and the response time was notably rapid at just 3 s. The sensor's exceptional sensing capabilities were attributed to its distinct urchin-shaped structure (Fig. 11G), which enhances oxygen activity and oxygen vacancy regulation. The sensor's performance was further assessed across various acetone concentrations, revealing a non-linear response relationship. This characteristic was significant in enabling the sensor to detect acetone effectively over a wide concentration range.

Xu et al. [85] fabricated 3D flower-like WO_3_ hierarchical structures for gas sensing applications, particularly focusing on ethanol detection. Their approach utilized NaHSO_4_ as a capping agent in a hydrothermal synthesis process, which enabled the manipulation of the specific surface area of the WO_3_ products. By varying the concentration of NaHSO_4_, they achieved control over the thickness and morphology of the nanosheets that composed the flower-like structures (Fig. 11H). The study found that increasing the NaHSO_4_ concentration from 6 g to 12 g resulted in a decrease in nanosheet thickness from approximately 30 nm to about 15 nm. The gas-sensing properties of these 3D flower-like hierarchical structured WO_3_ nanoparticles were evaluated by detecting different volatile gases at a lower concentration. The WO_3_ structures synthesized with 12 g of NaHSO_4_ demonstrated exceptional gas-sensitive properties, particularly towards ethanol. The sensitivity of these structures was as high as 96 under a concentration of 35 ppm at an optimal temperature of 350 °C.

## 5 Strategies for improving WO_3_ gas sensor performance

In addition to nanostructuring, various other approaches have been investigated to further enhance the gas sensing capabilities of WO_3_ based materials. These include crystal facet engineering, elemental doping, noble metal functionalization, heterojunction formation, composite development and UV activation.

### 5.1 Crystal facet engineering

WO_3_ demonstrates anisotropic crystal structure, with different facets exhibiting different surface energies and atomic arrangements. This results in varied chemical reactivity of the surfaces exposed on WO_3_ nanostructures.

In the research conducted by Wei et al. [87], the anisotropic crystal structure of WO_3_ was thoroughly investigated, revealing significant insights into the distinct facets and their influence on surface energies and chemical reactivity. The study successfully synthesized two unique morphologies of WO3: daisy-like hexagonal WO_3_ (h-WO_3_) and rose-like monoclinic WO_3_ (m-WO3), using a hydrothermal method. This methodological approach allowed for the creation of nanostructures with different crystal phases, providing an ideal platform for examining the activity of different crystal facets. The daisy-like h-WO_3_ consisted of radially oriented nanorods, resembling petals, and exhibited a dominant (002) facet exposure, as evidenced by the XRD patterns and TEM analysis. The chemical state analysis revealed insights into the electronic structure and surface composition of both h-WO_3_ and m-WO_3_. The findings suggested that h-WO_3_ possessed more oxygen vacancies and adsorbed oxygen species, indicative of more active adsorption sites, which could further enhance its sensitivity as a gas sensing material. Similar results were reported by other studies [43,44,[88], [89], [90]].

A study conducted by Gui et al. [91] demonstrated that the WO_3_ nanostructures primarily exposed (−112) facets, which were instrumental in achieving superior gas sensing performance and stability. This study is particularly relevant in highlighting the anisotropic nature of WO_3_. The findings indicate that the exposed (−112) facets of WO_3_, as opposed to other facets like (002) and (120), exhibit significantly higher chemical reactivity. This is evident from the enhanced sensing performance of these WO_3_ nanostructures towards triethylamine (TEA) at room temperature. The WO3 sensors with predominantly (−112) faceted surfaces showed a response approximately an order of magnitude higher than sensors with other exposed facets. This variation in response is a direct consequence of the different surface energies and atomic arrangements of the various facets. Additionally, the study utilized XPS and DFT calculations to further analyze the surface properties and chemical reactivity of the (−112) facets. The XPS data of one sample revealed the W_4f_ peaks were at binding energies of ∼37.1 eV and ∼35.0 eV, indicating W^5+^ oxidation state. In another sample, the W_4f_ peaks shifted to higher binding energies of 37.7 eV and 35.6 eV, matching W^6+^ oxidation state. This suggests the surface W cations are more oxidized. The shifting to higher oxidation state for W in a sample is supported by the presence of additional O 1s peaks at 532.0 eV related to adsorbed oxygen species. This indicates higher activity of the WO_3_ (−112) surface exposed in sample. This is corroborated by DFT calculations showing that the adsorption energy of TEA on the (−112) surface is significantly lower than on other facets, suggesting a stronger binding and hence a higher reactivity of the (−112) surface.

Yin et al. [92] revealed that the synthesis of thickness-controlled WO_3_ nanosheets successfully resulted in the formation of structures with exposed (020) and (200) facets. These facets exhibit distinct surface energies and atomic arrangements, which in turn influence the chemical reactivity of the surfaces. The research demonstrated that by altering the oxalic acid and HCl content in the preparation solutions, the average thickness of the WO_3_ nanosheets could be adjusted from approximately 10 to 110 nm. This adjustment in thickness and the resulting exposure of different facets significantly impacted the acetone sensing properties of the nanosheets. The findings showed a clear correlation between the exposure degree of (020) facets and the gas sensing performance. Specifically, WO_3_ nanosheets with a higher degree of exposed (020) facets exhibited enhanced acetone sensitivity and selectivity. This facet-dependent characteristic was a critical discovery, highlighting the role of surface structure in determining chemical reactivity. The research further postulated that the higher response to acetone could be attributed to the asymmetric arrangement of oxygen atoms on the exposed facets. This asymmetry potentially leads to a non-uniform distribution of the electron cloud over the surfaces, thereby influencing the local electric polarization on the exposed facets.

Song et al. [93] synthesized WO_3_ nanosheets in different phases, specifically monoclinic (M − WO_3_), triclinic (T-WO_3_), and hexagonal (H-WO_3_). Their research aimed to investigate the relationship between the crystal structure of WO_3_ and its gas-sensing performance, particularly towards NO_2_. Among them, T-WO_3_ nanosheets demonstrated superior gas-sensing performance with a high response, selectivity, and stability towards NO_2_ at low operating temperatures. This enhanced performance was attributed to the presence of more O_1c_ active sites on the main exposed crystal (200) facet of T-WO_3_, which facilitated the adsorption of NO_2_ molecules. Similar results were reported by other researchers [94].

Based on the research presented, it's evident that the study of WO_3_ in gas sensing applications is complex and multifaceted, with varying outcomes dependent on the specific crystal facets exposed. Different studies have explored how these facets, each with unique surface energies and atomic arrangements, influence WO_3_'s chemical reactivity and gas sensing performance. Despite these findings, there is no unanimous agreement within the scientific community regarding which facet or crystal structure of WO_3_ is most effective for gas sensing applications. The varied results across different studies underscore the complexity of WO_3_'s anisotropic nature and its impact on gas sensing performance. This ongoing research continues to evolve, with each study contributing to a deeper understanding of WO_3_'s properties and potential applications in gas detection technology.

### 5.2 Elemental doping

The incorporation of doping elements into WO_3_ gas sensors significantly enhances their performance. These elements introduce new electronic states, improving sensitivity and selectivity towards specific gases. Zhang et al. [95] developed mesoporous WO_3_ hollow nanospheres doped with varying concentrations of Fe. This innovation aimed at detecting low-level NO_2_ in environments ranging from air quality monitoring to asthma diagnosis through breath analysis. The Fe-doped WO_3_ nanospheres exhibited smaller cell parameters compared to pure WO_3_, suggesting a distortion in the crystal lattice that produced more defects beneficial for gas sensing. These nanospheres demonstrated a high crystalline quality and an exceptionally large surface area of approximately 165 m^2^/g. The study revealed that Fe doping led to an increase in oxygen vacancies in the WO_3_ structure, enhancing the adsorption of both oxygen and NO_2_, which is crucial for improved sensor performance. This enhancement was evident in the superior NO_2_ detection capabilities of the Fe-WO3 sensors, especially at low ppb-level concentrations. The sensors showed remarkable sensitivity, being able to detect NO2 concentrations as low as 10 ppb. Additionally, they exhibited a broad detection range (10–1000 ppb) and outstanding selectivity against other gases, making them highly efficient for specific NO_2_ detection. The optimum performance was observed in nanospheres with a 5.2% Fe concentration, which exhibited the best sensing capabilities at a relatively low operating temperature of 120 °C. Li et al. [96] focused on enhancing the gas sensing capabilities of WO_3_ nanofibers using Cr doping. The study revealed that the incorporation of Cr into WO_3_ nanofibers improved their xylene sensing performance. The gas-sensing tests showed that among the synthesized samples, the 4 mol% Cr-doped WO_3_ nanofibers exhibited the highest response to 100 ppm xylene. This response was about five times greater than that of pure WO_3_ nanofibers at an optimal operating temperature of 255 °C. The study proposed that the enhanced performance was likely due to increased oxygen vacancies, surface chemisorbed oxygen species, and lattice defects caused by Cr doping, leading to a higher charge carrier density and accelerated reactions with xylene. Yao et al. [97] synthesized microspheres of WO_3_ doped with Sb. By doping it with Sb, the research team was able to transform WO_3_ into a p-type semiconductor effective at near-room temperatures (25 °C–65 °C). This remarkable shift in properties was attributed to the change in the valency of tungsten (W) induced by Sb doping. At 35 °C, the sensor with 1 at% Sb-WO_3_ showed a detection limit of 200 ppb for NH_3_, along with high stability and selectivity against various potential interfering substances. This performance represents a significant improvement over traditional WO_3_ sensors, which require higher operating temperatures. Table 2 shows the performance of different elemental doping of WO_3_ for gas sensing.

**Table 2:** Sensing enhancement of WO_3_ nanostructures after doping with different element.

Doping element	Performance	Reference
Fe	Enabling the detection of NO_2_ at concentrations as low as 10 ppb, while maintaining a broad detection range of 10–1000 ppb and demonstrating superior selectivity.	[95]
	Fe-doping of WO_3_ sensors enhances their sensitivity to NO_2_ gas, with a increase in response amplitude at lower operating temperatures (150 °C), as evidenced by the Fe-doped WO_3_ film with 2.6 at% Fe showing a substantial sensitivity improvement towards 3–12 ppm NO_2_.	[98]
	Doping with 0.5 at% Fe increased the response of WO_3_ thin film gas sensors to 100 ppm CO by over 3 times at 150 °C compared to undoped WO_3_, with the response improving from 12% for WO_3_ to 40% for Fe-doped WO_3_.	[99]
	The Fe doping of WO_3_ increased the response to 10 ppm acetone from 1.2% for pure WO_3_ to 78% for Fe doped WO_3_ at 130 °C, as evidenced by the experimental data.	[100]
Ho^3+^	The 3 mol% Ho-doped WO_3_ sensor exhibited a 5-fold increase in acetone sensitivity compared to pure WO_3_, with maximum response of 15.2–100 ppm acetone at 200 °C.	[101]
In	The 5 wt% In-doped WO_3_ sensor exhibited an 11.2-fold increase in response to 50 ppm TEA at 115 °C compared to pure WO_3_.	[102]
Cr	Cr doping in WO_3_ sensors significantly enhances xylene detection, increasing the response by approximately five times to 35.04 for 100 ppm xylene at an optimal operating temperature of 255 °C, compared to undoped WO_3_ nanofibers.	[96]
	Cr doping in WO_3_ sensors enhances their efficiency, with a notable increase in formaldehyde sensing response from approximately 38% in undoped WO_3_ to around 82% in 1.5 at% Cr-doped WO_3_ at 200 °C for 50 ppm concentration in air	[103]
Sb	Sb doping transformed WO_3_ into an effective room-temperature NH_3_ sensor, achieving a detection limit of 200 ppb at 35 °C, with enhanced sensitivity and selectivity.	[97]
	The 3.5 wt% Sb-doped WO_3_ exhibited 9 times higher response to 8 ppm NO_2_ at 125 °C compared to pure WO_3_, as evidenced by gas sensing measurements.	[104]
Sn	Tin doping of WO_3_ nanosheets enhances alcohol sensing response, with 2% Sn-doped samples showing a 3–4 fold increase in response to 50 ppm methanol, ethanol and propanol compared to undoped WO_3_.	[105]
Ce	Ce doping in WO_3_ sensors enhances ethanol detection sensitivity, achieving a high response value of 12.3 for detecting 1 ppm ethanol, with a rapid response and recovery time of just 6 s.	[106]
Co	The introduction of Co doping in WO_3_ sensors enhances their sensitivity to acetone, with a 0.6 at% Co-doped WO_3_ sensor exhibiting a response approximately five times greater than that of a pure WO_3_ sensor to 100 ppm acetone gas.	[107]
	The doping of Co into WO_3_ enhances its ethanol gas sensing capabilities, as demonstrated by a notable increase in response values to different concentrations of ethanol, reaching as high as 2.339 for 20 ppm ethanol concentration in the case of 0.6% Co-doped WO_3._	[108]
	The incorporation of Co into WO_3_ sensors significantly enhances their acetone sensing capabilities, with the Co-doped WO_3_ exhibiting a high response value of 1.54 towards 1.5 ppm acetone at an operating temperature of just 50 °C, outperforming pure WO_3_ sensors.	[109]
Gd	Gd doping in WO_3_ nanostructures enhances the sensitivity of acetone sensors, with a 6 mol% Gd-doped WO_3_ exhibiting an optimal response of 27 towards 50 ppm acetone at an operating temperature of 350 °C, compared to undoped WO_3_.	[110]
	The inclusion of Gd in WO_3_/TiO_2_ nanocomposites resulted in a notable increase in sensor sensitivity, with the 3% Gd-doped composite showing the highest response towards NH_3_ gas, indicating a substantial improvement over undoped WO_3_ sensors	[111]
C	C doping enhances the sensitivity of WO_3_ sensors, evidenced by the fact that the sensor based on 3DOM C-doped WO_3_ with 410 nm pore size exhibits the highest responses to acetone, with responses increasing from 5.8 to 13.5 as the concentration of acetone increases from 0.9 ppm to 10 ppm.	[112]
	The C-doped WO_3_ MTs sensor exhibited an ultrahigh sensitivity with a response of 2.0–50 ppb toluene at the low operating temperature of 90 °C.	[113]
	C-doping of WO_3_ hollow nanospheres enhances the acetone sensing performance, as evidenced by the high sensitivity down to 0.2 ppm and excellent selectivity against other gases including ethanol, methanol, toluene, NH_3_, NO and CO.	[114]
Si	The Si-doping of the WO_3_ nanoparticles enhanced the sensor's sensitivity to acetone by 50% compared to undoped WO_3_, enabling accurate detection of acetone concentrations as low as 50 ppb.	[115]

### 5.3 Noble metal functionalization

Decorating WO_3_ surface with noble metals like Pt, Pd and Au has been extensively used to dramatically improve its gas sensing performance. The noble metal nanoparticles act as catalysts to accelerate the dissociation of oxygen molecules into more reactive atomic species. This increases the concentration of chemisorbed oxygen on the WO_3_ surface available for reaction with target gas molecules.

The incorporation of Ag into WO_3_ was found to significantly improve the WO_3_ towards NO detection [116]. The experiment involved preparing WO_3_ powder with 1% Ag doping and analyzing its performance in sensing NO gas. The results showed a dramatic increase in sensitivity compared to undoped WO_3_, especially at lower temperatures. The optimal sensor temperature for detecting NO was reduced from the standard 300 °C (common for most WO_3_ sensors) to below 200 °C due to Ag doping. This decrease in operational temperature, accompanied by enhanced sensitivity, marked a significant improvement in sensor performance and efficiency. Ag doping did not alter the bulk structure of WO_3_. However, it was found to create a high concentration of oxygen vacancies, forming coordinated crystallographic shear planes on the WO_3_. This structural modification at the Ag-WO_3_ interface was proposed as a key factor in the enhanced sensitivity. The researchers hypothesized that the Ag particles facilitated the oxidative conversion of NO to NO_2_, with subsequent adsorption on the defective WOx sites at the Ag-WO_3_ interface. Au fictionization has been used as well. Researchers investigated the enhancement of NO_2_ gas detection capabilities using Au-WO_3_ sensors [117]. The study found that Au-WO_3_ significantly improved the gas sensing properties. Specifically, the 1.0 wt% Au-WO_3_ sensor demonstrated a larger response, better selectivity, faster response/recovery times, and longer-term stability to NO_2_ at a lower operating temperature (150 °C) compared to its undoped counterpart. This enhanced performance was attributed to the increased active sites and altered charge state induced by the Au fictionization, which facilitated more effective interactions with NO_2_ molecules. The research also revealed that while the size of the WO_3_ particles remained unaffected by Au fictionization, the size of the Au particles within the doped WO_3_ had a notable impact on sensor performance. Larger Au particles were found to be less effective, suggesting that smaller Au particles might lead to further improvements in gas response. Fardindoost et al. [118] explored the enhancement of hydrogen gas sensing capabilities using WO_3_ films doped with Pd. A pivotal aspect of this research was investigating how varying concentrations of Pd affected the properties and performance of the WO_3_ films. The study revealed that the inclusion of Pd notably influenced the growth kinetics and crystallite size of WO_3_ nanoparticles. Specifically, an increase in Pd concentration resulted in a decrease in the crystallite size of WO_3_, which was instrumental in enhancing the gas sensitivity of the films. This reduction in crystallite size was attributed to the likelihood of Pd particles accumulating at the grain boundaries of WO_3_, thereby impeding grain growth during heat treatment. Crucially, the Pd-doped WO_3_ films demonstrated a significant improvement in hydrogen gas sensitivity compared to pure WO_3_ films. The films exhibited a remarkable sensitivity of about 2.5 × 10^4^ at room temperature for hydrogen concentrations of 0.1% in air. Notably, the optimum working temperature for these sensors was identified to be around 100 °C, balancing both sensitivity and response time. Additionally, the study observed that higher concentrations of Pd not only increased sensitivity across a wide temperature range but also reduced the sensor's operating temperature. However, it was also noted that the recovery time for the sensors, especially at room temperature, was an area requiring further optimization. Table 3 summarizes the change of gas sensing performance of WO_3_ after fictionization of noble metals.

**Table 3:** Sensing enhancement of WO_3_ nanostructures after fictionization with different noble metals.

Nobel metal	Performance	Reference
Ag	The introduction of Ag doping into WO_3_ sensors improved their sensitivity to NO gas, achieving a sensitivity increase up to 21.5 at 300 °C, compared to 7.1 for undoped WO_3_.	[116]
	Ag doping in WO_3_ nanofiber sensors enhances NO_2_ sensing performance, evidenced by a 3 mol% Ag-doped sample achieving a gas response of 90.3 at 225 °C, approximately nine times higher than the 12.0 response of the undoped WO_3_ sensor at 250 °C.	[119]
Au	With the 1.0 wt% Au-doped sensor showing a notably larger response and improved selectivity to NO_2_ gas, particularly at a lower operating temperature of 150 °C, compared to the undoped variant.	[117]
	The 0.30 at% Au-doped WO_3_·H_2_O sensor exhibited a 26.4-fold higher response to 5 ppm xylene compared to undoped WO_3_·H_2_O, owing to the catalytic activity of Au nanoparticles.	[120]
	The NO_2_ response of the Au-doped WO_3_ microspheres was enhanced by over 5 times compared to the pure WO_3_ microspheres at the optimal operating temperature.	[121]
Pd	Pd doping in WO_3_ sensors notably elevated hydrogen gas sensitivity to approximately 25,000 times higher at room temperature, compared to undoped films, demonstrating enhanced detection capabilities for hydrogen concentrations as low as 0.1% in air.	[118]
	Pd doping in WO_3_ sensors significantly enhances their xylene sensing performance, increasing the response value to 21.0 for 10 ppm xylene at an optimal operating temperature of 230 °C, compared to a much lower response in undoped WO_3_.	[122]
	The doping of Pd into WO_3_ sensors enhances hydrogen gas detection efficiency, elevating the sensor response from 1.07 to 11.78, as demonstrated by the improved performance of 1 wt% Pd-doped mesoporous WO_3_ compared to undoped WO_3_.	[123]
Pt	Pt doping in WO_3_ sensors leads to a significant shift in work function, with measurements showing an increase from 5.013 eV in undoped WO_3_ to 5.126 eV in WO_3_ doped with Pt, indicating altered electronic properties and enhanced gas sensing capabilities."	[39]
	Pt doping enhanced the NH_3_ gas sensing performance of the WO_3_-based sensors, with 1.0 mol% Pt-doped WMSs exhibiting a 4 times higher response to 1000 ppm NH_3_ at 175 °C compared to pure WMSs.	[124]
Ru	The Ru doping of WO_3_ led to a 32.5% increase in acetone sensitivity compared to pure WO_3_, as evidenced by the gas sensing measurements.	[125]
	The gas response of the 0.5 wt% Ru-WO_3_ sensor towards 100 ppm xylene increased from 11 to 73 compared to pure WO_3_, demonstrating the significant enhancement provided by Ru doping.	[126]
	The trace loading of Ru (0.01–0.02 wt%) on WO_3_ nanoparticles resulted in a 100-fold enhancement in sensor response to 0.5 ppm acetone.	[127]
Rh	The addition of 1 wt% Rh increased the sensor response of WO_3_ nanosheets to 5 ppm acetone gas from 1.2 to 28 at 300 °C.	[128]
	Rh decoration provided a 100-fold enhancement in acetone sensitivity for the WO_3_ nanorods, with a detection limit as low as 10 ppb.	[129]
	The Rh-loaded WO_3_ sensor achieved a 2 s response time, 40 ppb detection limit, and 80% higher acetone response at 80% Rh compared to pure WO_3._	[130]

### 5.4 Heterojunction formation

Coupling WO_3_ with n-type (ZnO, SnO_2_, TiO_2_) or p-type (NiO, CuO) semiconducting oxides results in the formation of n-n or p-n heterojunctions. This leads to electron transfer across the interface until the Fermi levels align. The band bending creates charge depletion regions which act as potential barriers inhibiting electron transport.

**Fig. 12:**
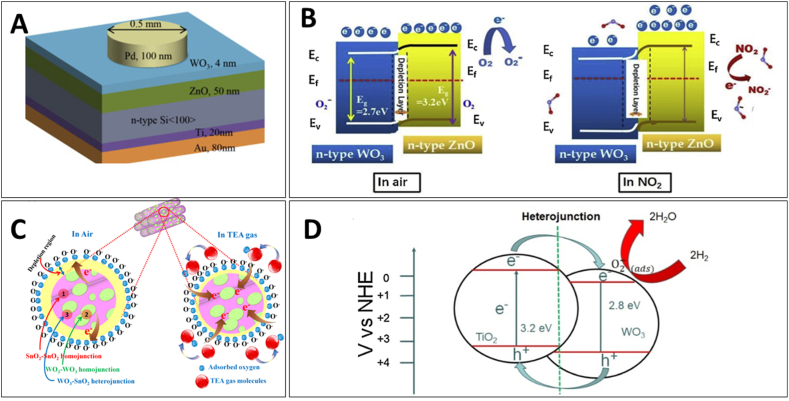
(A) Schematic cross section of Pd/WO_3_/ZnO/Si Schottky diodes [131]. (B) Energy band structures of WO_3_ and ZnO [132]. (C) Formation of depletion layer for WO_3_–SnO_2_ material [133]. (D) Scheme of hydrogen sensing mechanism of WO_3_–TiO_2_ composite based on heterojunction effect [134]. Permission obtained from ELSEVIER.

Implementation of a WO_3_/ZnO heterojunction between the Pd contact and Si substrate in the Schottky diode hydrogen sensor has been investigated [131]. This heterojunction structure provides several important benefits for improving the sensor performance. Firstly, the WO_3_ grown on ZnO has a rougher surface morphology and more stoichiometric composition, which increases the effective surface area and provides more hydrogen adsorption sites. Secondly, the WO_3_/ZnO interface forms an additional potential barrier which increases the effective Schottky barrier height. This barrier height modulation enhances the sensor's sensitivity to changes in surface charge induced by hydrogen adsorption. As a result, the Pd/WO_3_/ZnO/Si (Fig. 12A) sensor demonstrates a 10 times higher voltage response, faster response/recovery kinetics, and ability to detect hydrogen at lower concentrations compared to the standard Pd/WO_3_/Si diode. In another work [132], WO_3_/ZnO nanocomposites with n-n isotype heterojunctions has been prepared by loading ZnO onto mesoporous WO_3_ nanocrystals. The addition of ZnO and formation of WO_3/_ZnO heterojunctions significantly improved the gas sensing performance of mesoporous WO_3_ to NO_2_. Specifically, the 5 wt% ZnO/WO_3_ sensor exhibited much higher response and better selectivity to NO_2_ compared to pure mesoporous WO_3_. The enhanced sensing performance is attributed to the n-n heterojunctions formed at the interface between WO_3_ and ZnO nanocrystals. The heterojunctions lead to bending of energy bands and formation of a depletion layer (Fig. 12B), which increases resistance and improves gas sensitivity. Moreover, the heterojunctions can facilitate charge transfer and separation of electron-hole pairs, further increasing sensor response. In summary, the WO_3_/ZnO heterojunctions play a critical role in enhancing the response, selectivity and stability of the mesoporous WO_3_ sensor for NO_2_ detection. The synergistic effect between WO_3_ and ZnO through n-n heterojunction formation is essential for achieving excellent gas sensing performance.

The formation of WO_3_–SnO_2_ heterojunctions also been widely investigated. The junction between the two metal oxides with different work functions leads to transfer of electrons from SnO_2_ to WO_3_ until the Fermi levels align. This creates a depletion region and band bending at the interface, causing a built-in potential. When exposed to TEA gas, electron donation from the gas molecules to the oxide surface leads to changes in the depletion region width and band bending (Fig. 12C) [133]. This greatly amplifies the sensor's response through modulation of the heterojunction resistance. Specifically, the WO_3_–SnO_2_ heterojunction enables higher sensitivity, faster response/recovery kinetics, and lower operating temperature compared to pure SnO_2_ and WO_3_ sensors. The sensitivity is improved by the junction's electronic sensitization effect which produces a much larger change in resistance upon gas exposure. The response/recovery is accelerated by faster diffusion and migration of gas molecules and electrons within the heterojunction. And the operating temperature is reduced due to the lower activation energy of the heterojunction-enhanced sensing mechanism. In another work [135], hollow structured WO_3_–SnO_2_ composites showed superior sensing behaviors compared to the solid ones, including higher sensitivity, faster response and recovery, and better selectivity for acetone. For example, the hollow structured composites demonstrated a sensitivity of 21.2 to 5 ppm acetone versus 11.3 for the solid composites.

Li et al. [134] investigated a WO_3_–TiO_2_ heterojunction for developing a room temperature hydrogen gas sensor. The heterojunction formed between the two metal oxides introduces a built-in potential and interfacial defects, which can facilitate electron transfer and band bending when exposed to hydrogen gas (Fig. 12D). This enables efficient hydrogen adsorption and desorption on the material surface, leading to significant variation in electrical resistance that signals the presence of hydrogen. Specifically, the WO_3_–TiO_2_ heterojunction sensor demonstrated a high response of 5.26–10,000 ppm H_2_ along with short response and recovery times of 10 s and 5 s, respectively. This is a major improvement over the pure TiO_2_ sensor which required 1974 s for a complete cycle. The heterojunction provides a synergistic effect that enhances the kinetics for hydrogen sensing. In another work [136], researchers developed a mixed potential NH_3_ sensor using a TiO_2_@WO_3_ core-shell composite as the sensing electrode. The key finding was that the sensor with the TiO_2_@WO_3_ composite electrode demonstrated greatly improved NH_3_ sensing capabilities compared to sensors using TiO_2_, WO_3_, or a TiO_2-_WO_3_ mixture as the electrode. At 450 °C, the sensor achieved a maximum NH_3_ sensitivity of 74.8 mV/decade. The TiO_2_-WO_3_ heterojunction modulated the electrical transport properties.

**Fig. 13:**
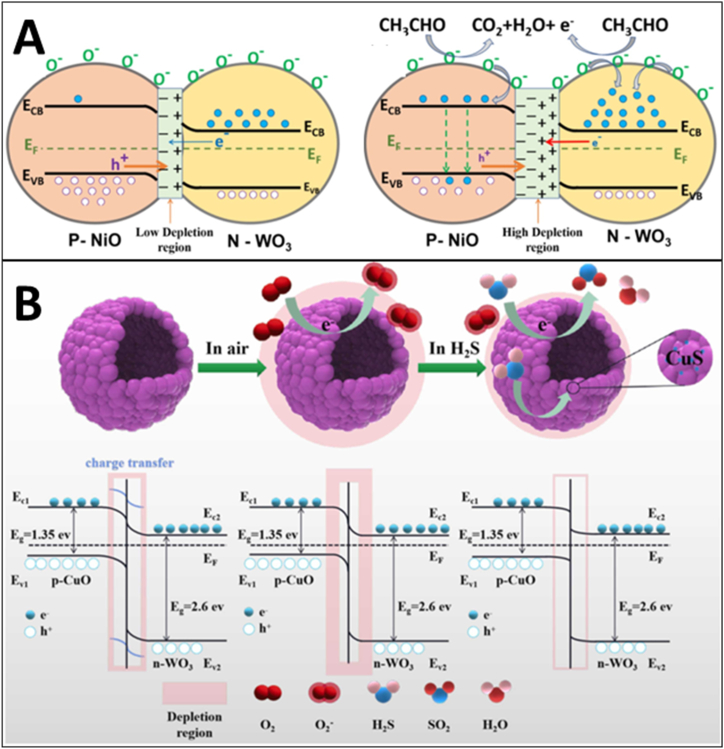
(A) Band diagram for NiO/WO3 interface in air and after interface in target gas [137]. (B) Schematic of the H_2_S gas sensing mechanism on the CuO/WO_3_ hollow microspheres at 70 °C [139]. Permission obtained from ELSEVIER.

A p-n heterojunction between p-type NiO nanosheets and n-type WO_3_ nanorods plays a critical role in improving the acetaldehyde gas sensing performance compared to the individual NiO and WO_3_ components [137]. Specifically, the p-n junction leads to band bending and the formation of a built-in potential, which facilitates electron transfer from the conduction band of n-type WO_3_ to the valence band of p-type NiO. This transfer of electrons across the heterojunction interface greatly increases the sensor's response to acetaldehyde gas (Fig. 13A). Additionally, the high density of interface states in the depletion region acts as preferential sites for oxygen adsorption and reactions with acetaldehyde gas molecules. This further enhances the sensor's response through modulating its electrical resistance. Gao et al. [138] developed a novel gas sensor based on hollow WO_3_–NiO nanoflowers for fast and selective detection of xylene. Comprehensive gas sensing tests showed that this sensor exhibited exceptional xylene sensing capabilities. Specifically, it demonstrated ultrahigh sensitivity to xylene down to 1.5 ppb along with short response and recovery times within 1 min. At the optimal 300 °C, the xylene sensitivity was 8.1 and 10.3 times higher than acetone and ethanol. The researchers attributed the remarkable performance to the unique hollow porous morphology and p-n heterojunctions formed between WO_3_ and NiO.

The formation of p-n heterojunctions between p-type CuO and n-type WO_3_ plays an important role in enhancing the H_2_S sensing performance [140]. When the two semiconducting oxides come into contact, a depletion region is formed at the interface due to the diffusion of charge carriers. This creates a built-in potential barrier that resists the further flow of carriers. However, in the presence of a reducing gas like H_2_S, the oxides are converted to sulfides, which changes their conductivity. This leads to a destruction of the p-n heterojunctions and a large decrease in resistance of the sensor film. The p-n junctions also help improve the response and recovery kinetics of the sensor by providing rapid diffusion paths for the gas molecules. Overall, the creation of p-n heterojunctions between CuO and WO_3_ increases the H_2_S response, lowers the operating temperature, and speeds up the response and recovery times of the sensor films. Wang et al. [139] reported a similar work recently. The team first synthesized hollow microspheres of WO_3_ by dissolving tungsten chloride in acetic acid and hydrothermally treating the solution. The resulting precursor was washed, dried, and annealed to obtain the WO_3_ powder. To make the CuO/WO_3_ composite, copper nitrate was added during the initial dissolution step. Characterization showed that while the CuO/WO_3_ retained the hollow microsphere structure of WO_3_, it had smaller diameters and thicker shells. Gas sensing tests revealed the CuO/WO_3_ composite had far superior performance to WO_3_ alone. At the optimal temperature of 70 °C, the CuO/WO_3_ sensor gave a response of 1297 to 10 ppm H_2_S, around 103 times higher than pure WO_3_. This dramatic improvement was attributed to the p-n heterojunction formed between p-type CuO and n-type WO_3_, as well as the sulfurization of CuO to CuS in the presence of H_2_S (Fig. 13B). The CuO/WO_3_ sensor also demonstrated rapid response, lower detection limits down to 100 ppb, excellent selectivity, and continuous cycle H_2_S detection from 0.1 to 50 ppm.

### 5.5 Hybrid nanocomposites

Creating hybrids of WO_3_ with graphene (GR), carbon nanotubes, conductive polymers or organic compounds results in composite interfaces that promote electron transfer between the constituents. This enhances charge carrier concentration and mobility.

**Fig. 14:**
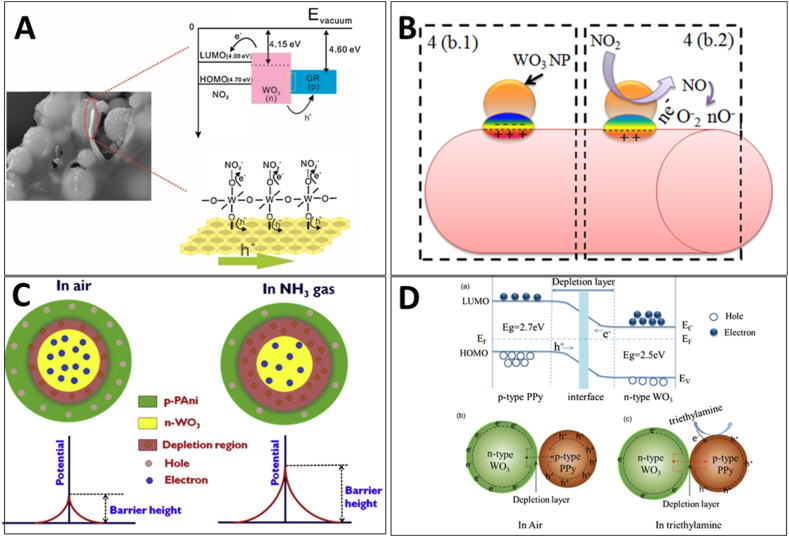
(A) Sensing mechanism of GR-WO_3_ composites to NO_2_ at room temperature and electron transfer between WO_3_ nanospheres and graphene sheets [141]. (B) Schematic illustration of the NO_2_ sensing mechanisms of MWCNTs-WO_3_ [143]. (C) Sensing model of flexible PAni-WO_3_ hybrid nanocomposite sensor when exposed to air and NH_3_ gas [144]. (D) The energy band structure and schematic model for the PPy/WO_3_ heterojunction based sensor [145]. Permission obtained from ELSEVIER.

A study synthesized GR-wrapped WO_3_ nanosphere composites using a facile sol-gel method in order to develop room temperature NO_2_ gas sensors [141]. The composites exhibited uniform nanospheres with diameters of 200–400 nm. Unlike pure WO_3_ nanoplates and graphene sensors, the GR-wrapped WO_3_ nanocomposite sensors demonstrated good response and selectivity to low concentrations of NO_2_ gas at room temperature. Specifically, upon exposure to 56 ppm NO_2_, the GR-WO_3_ sensor's response reached 40.8%, while the pure WO_3_ and graphene sensors showed no responsiveness. The researchers proposed that the effective charge transfer occurring through the chemically bonded interfacial contact between the graphene sheets and WO_3_ nanospheres was responsible for enabling the room temperature sensing performance (Fig. 14A). The combination of the high specific surface area of graphene and the sensitivity of WO_3_ facilitated gas diffusion and enhanced the gas chemisorption reaction. The results of this study highlight a simple method to synthesize GR-WO_3_ nanocomposites with unique nanostructures tailored for room temperature NO_2_ gas sensing applications. Reduced graphene oxide (RGO) has also been used for incorporating with WO_3_ for NO_2_ sensing [142]. The researchers combined a one-pot polyol process with a metal organic decomposition method to produce the RGO/WO_3_ nanocomposite films. The researchers systematically studied how the amount of RGO affected the electrical and NO_2_ gas sensing properties of the films at room temperature. They found that incorporating an optimal amount of RGO into the WO_3_ film markedly improved the response and sensitivity to NO_2_ gas at room temperature compared to a pure WO_3_ film. The RGO provided a conductive network and increased the surface area. This enhanced the gas interaction and electron transfer processes, allowing for effective NO_2_ detection at low temperatures.

Yaqoob et al. [143] developed and tested a flexible NO_2_ gas sensor fabricated using a hybrid material of multi-walled carbon nanotubes (MWCNTs) and WO_3_. The researchers aimed to create a lightweight, robust NO_2_ sensor that could maintain performance even when flexed or bent. The sensor demonstrated a maximum response of 14% when exposed to 5 ppm NO_2_, with a low limit of detection of 0.1 ppm. The addition of WO_3_ to the MWCNTs helped improve the recovery time to baseline after NO_2_ exposure compared to pure MWCNT sensors. The researchers suggested the MWCNTs provided the flexible scaffolding and high conductivity needed for flexible gas sensing, while the WO_3_ offered extra surface area and catalytic sites to improve sensitivity and recovery (Fig. 14 B).

Many conductive polymers were used for enhancing the sensing performance of WO_3_. Among them, polyaniline (PAni) is a good choice. For example, researchers developed a flexible NH_3_ gas sensor using PAni-WO_3_ hybrid [144]. The gas sensing performance of the flexible PAni-WO_3_ sensors was evaluated and compared to pure PAni and WO_3_ sensors. It was found that the PAni-WO_3_ sensor with 50 wt% WO_3_ loading exhibited optimal response, with 121% change in resistance when exposed to 100 ppm NH_3_ at room temperature. This was a significant improvement over the pure PAni and WO_3_ sensors. The hybrid sensor also demonstrated a low detection limit of 1 ppm NH_3_, with 9% response. In addition to high sensitivity, the flexible PAni-WO_3_ sensor showed excellent selectivity towards NH_3_ over other gases like CO_2_ and ethanol. The sensing mechanism of the flexible PAni-WO_3_ hybrid sensor involves protonation interactions between NH_3_ gas and the PAni-WO_3_ composite. When the sensor is exposed to NH_3_, the lone pair of electrons on the N atom of NH_3_ donates protons to the N atoms of PAni. This protonation process interrupts the conjugation in PAni chains, increasing charge carrier scattering which increases the resistance of the PAni-WO_3_ composite. The WO_3_ nanoparticles in the hybrid enhance the protonation effect, leading to greatly improved NH_3_ sensitivity compared to pure PAni sensors (Fig. 14C). Overall, NH_3_ exposure causes an increase in resistance which is measured as the sensor response.

Polypyrrole (PPy) is another widely studied conductive polymer. Researchers developed a flexible and portable gas sensor using PPy and WO_3_ nanoparticles to detect TEA at room temperature [145]. The sensor was fabricated by depositing PPy/WO_3_ hybrids made through in situ chemical oxidation polymerization onto a polyethylene terephthalate film substrate. The results showed that WO_3_ nanoparticles were evenly distributed in the PPy matrix. When tested for TEA sensing, the PPy/WO_3_ sensor exhibited a response of 680% to 100 ppm TEA at room temperature, much higher than other reported PPy hybrid sensors. The high sensitivity was attributed to the complementary effect and formation of a p-n heterojunction between p-type PPy and n-type WO_3_. This heterojunction improved charge transfer and gas diffusion to boost sensor performance (Fig. 14D).

The complementary properties of the composite components facilitate electronic and chemical sensitization which amplifies the gas response. The composites also benefit from increased porosity, surface area and active sites.

### 5.6 UV activation

**Fig. 15:**
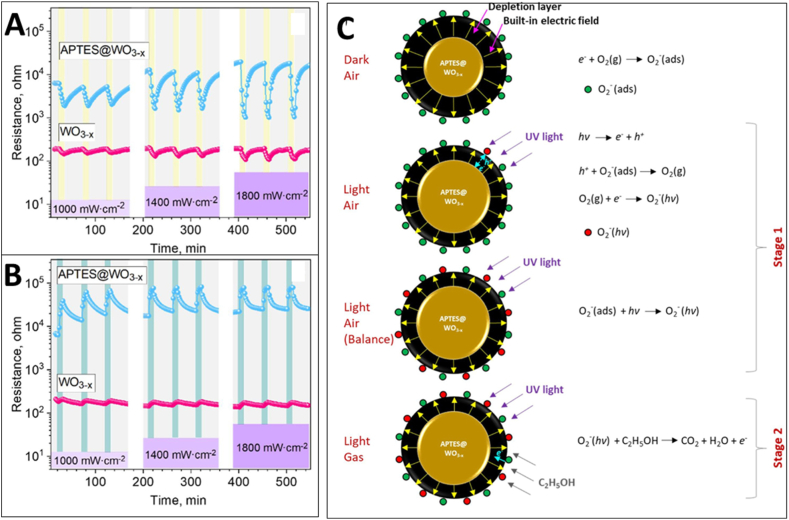
Typical resistance changes for the sensors based on WO_3-x_ and APTES@WO_3-x_ sensors to 80 ppm of (A) ethanol and (B) NO_2_ and various radiant flux. (C) Schematic illustration of the gas sensing mechanism of UV-LED-activated APTES@WO_3-x_ sensor under different conditions [147]. Permission obtained from ELSEVIER.

Illuminating WO_3_ with UV light generates photogenerated electron-hole pairs which increase carrier concentration and reduce band bending. Visible light can also excite electrons across the narrow bandgap of WO_3_. This makes desorption of target gas molecules easier under light, improving response and recovery kinetics. Bouchikhi et al. [146] applied UV light irradiation at 394 nm to WO_3_ nanowire gas sensors, both pristine and decorated with metal nanoparticles, for formaldehyde detection. It was found that UV irradiation significantly reduced the response and recovery times for formaldehyde gas compared to operation under dark conditions. This demonstrates that the UV light provides additional energy that accelerates the adsorption and desorption processes occurring on the WO_3_ surface during gas sensing. Faster response and recovery enables more sensitive real-time monitoring of formaldehyde concentrations. Furthermore, UV irradiation was shown to diminish the baseline drift typically observed with metal oxide gas sensors like WO_3_. This baseline shift is caused by strong chemical adsorption of gases, but the extra energy from UV illumination helps desorb these species. Reducing baseline drift improves the stability and repeatability of measurements. A recent study investigated the gas sensing properties of WO_3-x_ nanowires that were modified with (3-aminopropyl)triethoxysilane (APTES) [147]. The researchers integrated the APTES-modified WO_3-x_ nanowires into microsensors and tested their ability to detect gases under UV light activation at room temperature. The results demonstrated that the APTES@WO_3-x_ sensors had enhanced sensitivity and selectivity to certain gases compared to unmodified WO_3-x_ sensors. Specifically, the APTES@WO_3-x_ sensors displayed approximately 17 times higher sensitivity to ethanol vapor (Fig. 15A) and 20 times higher sensitivity to nitrogen dioxide gas relative to the non-modified WO_3-x_ sensors when activated by UV light. The APTES@WO_3-x_ sensors also showed improved selectivity towards sensing NO_2_ (Fig. 15B). The researchers attributed the superior gas sensing performance of the APTES@WO_3-x_ sensors to the presence of an amino group on the APTES molecule. They proposed that this amino group facilitated chemical interactions and electron transfer between the target gas molecules and the WO_3-x_ nanowires when the sensors were activated by UV light. The UV excitation provided sufficient energy to promote charge carriers in the WO_3-x_ nanowires, while the amino group on APTES served as a reactive site to bind gas molecules and influence the electrical properties of the WO_3-x_. The combined effect led to the enhanced sensitivity and selectivity of the APTES@WO_3-x_ sensors towards certain gases under UV activation (Fig. 15C).

## 6 Challenges and future outlook

Although nanostructuring and composites have enhanced WO_3_ sensor sensitivity into the ppb range for gases like NO_2_, NH_3_ and VOCs, further improvements would enable new applications in medical diagnostics, environmental safety, and industrial hygiene. Future work could involve engineering 3D WO_3_ morphologies with higher porosity for maximum surface area and gas accessibility. Novel synthesis methods like flame spray pyrolysis allow large scale production of pure and doped WO_3_ nanoparticles without agglomeration, which could help improve response. Decorating ultra-thin 2D WO_3_ nanosheets with smaller and well dispersed metal nanoparticles can further boost sensitivity. Machine learning models could help determine optimal configurations.

WO_3_ sensors tend to show cross-sensitivity to different gases especially at higher temperatures, limiting selective detection. This could be mitigated through statistical analysis of data from sensor arrays. Doping with transition metals like Cr, Ti, Fe that interact preferentially with certain gases could tune selectivity. Lowering operating temperatures also enhances selectivity. Response transients analysis provides chemical fingerprinting for identification. Long term stability is affected by factors like grain growth, sintering and volatility/leaching of dopants at elevated temperatures. This could be improved through better encapsulation and surface functionalization. Aging studies under realistic environments are needed. Doping with retention-enhancing elements needs more focus. Reversible operation between room temperature and higher temperatures may provide self-healing while avoiding permanent degradation.

High ambient humidity leads to moisture adsorption on WO_3_, affecting sensitivity and stability. Composite materials like graphene exhibit humidity shielding effects and could help mitigate this interference. Surface functionalization with hydrophobic groups needs more exploration. Operation at lower temperatures reduces moisture effects but may impact kinetics. The time for sensor resistance to return to baseline on gas removal is relatively long, especially at room temperature. This could be accelerated by newer nanostructures providing low desorption energies. Plasmonic metal nanoparticles facilitate photo-desorption upon light exposure following gas response. Applied thermal pulses can provide energy for faster desorption. Circuit techniques like AC modulation aid faster baseline recovery.

Most WO_3_ sensors require temperatures of 200–400 °C for optimal performance. This leads to higher power consumption. Using advanced materials like CuO nanowires or graphene to form heterojunctions lowers the operating range closer to room temperature. Light assisted operation also activates WO_3_ at lower temperatures. New transduction principles like capacitance, FET or piezoelectric modes could also reduce temperature needs. The requirement for temperature control and electrode interfacing poses integration challenges especially for portable platforms. Emerging micro-hotplate designs incorporated with CMOS circuitry enable on-chip integration of sensing layers. Use of temperature pulses for short durations may assist mobile applications. Wireless interrogation methods avoid complex sensor wiring. Flexible and miniaturized platforms need focus.

While nanostructured WO_3_ sensors have seen remarkable progress, continuous innovation is needed to address intrinsic material limitations and emerging application needs. Key research gaps exist in areas such as multifunctional nanocomposite development, real-world deployment challenges, and long-term stability assessments. Specifically, engineering multifunctional WO_3_ nanocomposites with other metal oxides or graphene could provide opportunities to further tune sensitivity, selectivity and response kinetics. However, real-world integration and field testing of such sensor materials needs more focus. Robust packaging solutions and wireless interrogation methods are necessitated for applications in wearable platforms and wireless sensor networks. In addition, stability and drift issues affected by factors like grain growth, sintering and dopant volatility need to be rigorously characterized through long-term aging studies across operating temperatures and humid ambient conditions. Such reliability assessments would help identify critical failure mechanisms and guide future materials enhancement strategies.

## 7 Conclusions

The development of nanostructured WO_3_ materials has ushered remarkable advancements in gas sensor technology over the past decade. The ability to precisely control morphology at the nanoscale has led to significant improvements in sensitivity, response time, and limit of detection across a wide variety of gases. Engineered 1D structures like nanorods and 0D nanoparticles have provided extremely high surface area to volume ratios for maximizing gas accessibility and reactivity. The exposure of unique crystal facets in anisotropic nanostructures has offered new avenues for selectivity tuning. Additionally, elemental doping, noble metal functionalization and heterostructure formation have further enhanced the gas interaction pathways and charge transport kinetics. Novel 3D assemblies exhibit optimal combinations of porosity, interconnectivity and surface activity. The progress has been accelerated by facile and scalable synthesis techniques that allow morphology, dopant and composite control. Cumulatively, these nanoengineering strategies have boosted WO_3_ sensor performance to parts per billion levels for gases like NH_3_, NO_2_ and VOCs. However, for reliable real-world deployment across environmental, industrial and biomedical applications, key reliability challenges around selectivity and stability need mitigation. Cross-sensitivity issues are being addressed through multivariate data analysis, temperature modulation and doping optimization. Long term drift arising from sintering, grain growth and dopant variations necessitates robust encapsulation and surface passivation techniques. In addition, humidity tolerance remains a persistent problem. Despite remarkable sensitivity feats, most WO_3_ sensors continue to operate in the 200–500 °C range, causing integration difficulties especially for portable devices. Tackling these limitations and unlocking new possibilities requires an interdisciplinary approach combining materials innovation, device engineering and data analytics. Developing mutlifunctional nanocomposites, assessing failure modes under realistic conditions and modeling property-performance correlations can accelerate future advancement. With its inherent stability, ease of fabrication and versatility for composition tuning, nanostructured WO_3_ remains well poised to drive innovation in gas sensor systems across diverse application domains.

## Data availability statement

No data was used for the research described in the article.

## CRediT authorship contribution statement

**Xingxing Li:** Investigation, Data curation. **Li Fu:** Writing – review & editing, Conceptualization. **Hassan Karimi-Maleh:** Writing – review & editing, Methodology, Conceptualization. **Fei Chen:** Methodology, Investigation. **Shichao Zhao:** Supervision, Resources, Project administration.

## Declaration of competing interest

The authors declare that they have no known competing financial interests or personal relationships that could have appeared to influence the work reported in this paper.
